# Long noncoding RNA MAPKAPK5-AS1 promotes colorectal cancer progression by cis-regulating the nearby gene MK5 and acting as a let-7f-1-3p sponge

**DOI:** 10.1186/s13046-020-01633-8

**Published:** 2020-07-20

**Authors:** Ting Yang, Wei-Cong Chen, Pei-Cong Shi, Man-Ru Liu, Tao Jiang, Hu Song, Jia-Qi Wang, Rui-Zhi Fan, Dong-Sheng Pei, Jun Song

**Affiliations:** 1 Department of General Surgery, The Affiliated Hospital of Xuzhou Medical University, Xuzhou, 221002 Jiangsu Province China; 2 Department of Pathology, Xuzhou Medical University, Xuzhou, 221002 Jiangsu Province China; 3 Institute of Digestive Diseases of Xuzhou Medical University, Xuzhou, 221002 Jiangsu Province China

**Keywords:** MK5-AS1, MK5, Let-7f-1-3p, SNAI1, CRC

## Abstract

**Background:**

Long noncoding RNAs (lncRNAs) are considered critical regulators in cancers; however, the clinical significance and mechanisms of MAPKAPK5-AS1 (hereinafter referred to as MK5-AS1) in colorectal cancer (CRC) remain mostly unknown.

**Methods:**

In this study, quantitative real-time PCR (qPCR) and western blotting were utilized to detect the levels of MK5-AS1, let-7f-1-3p and MK5 (MAPK activated protein kinase 5) in CRC tissues and cell lines. The biological functions of MK5-AS1, let-7f-1-3p and MK5 in CRC cells were explored using Cell Counting Kit-8 (CCK8), colony formation and transwell assays. The potential mechanisms of MK5-AS1 were evaluated by RNA pull-down, RNA immunoprecipitation (RIP), dual luciferase reporter assay, chromatin immunoprecipitation (ChIP) and bioinformatics analysis. The effects of MK5-AS1 and MK5 on CRC were investigated by a xenotransplantation model.

**Results:**

We confirmed that MK5-AS1 was significantly increased in CRC tissues. Knockdown of MK5-AS1 suppressed cell migration and invasion in vitro and inhibited lung metastasis in mice. Mechanistically, MK5-AS1 regulated SNAI1 expression by sponging let-7f-1-3p and *cis*-regulated the adjacent gene MK5. Moreover, MK5-AS1 recruited RBM4 and eIF4A1 to promote the translation of MK5. Our study verified that MK5 promoted the phosphorylation of c-Jun, which activated the transcription of SNAI1 by directly binding to its promoter.

**Conclusions:**

MK5-AS1 *cis*-regulated the nearby gene MK5 and acted as a let-7f-1-3p sponge, playing a vital role in CRC tumorigenesis. This study could provide novel insights into molecular therapeutic targets of CRC.

## Background

Colorectal cancer (CRC) is the third most common cancer among males and the second most common cancer among females, with high incidence and high mortality [1]. Although, cancer biomarkers, surgical excision, radiotherapy and chemotherapy [2] have been applied to CRC diagnosis and treatment, patients have a poor prognosis. Recurrence, metastasis and drug resistance [3] are the main causes of poor prognosis in patients with CRC. The aggressive colorectal neoplasm has complex biological characteristics, involving a series of pathophysiological changes, as well as aberrant regulation of genes and signaling pathways [4, 5]. Therefore, effective tumor markers and therapeutic targets for CRC are of paramount importance.

Long noncoding RNAs (lncRNAs) are a class of noncoding RNAs with a length greater than 200 nucleotides [6] and are categorized based on regulating gene expression in *cis* versus performing functions in *trans* [7]. In recent years, emerging evidence has shown that lncRNAs play major roles in the occurrence and development of cardiovascular disease [8], cardiomyopathy [9] and mesenchymal stem cells [10, 11], in particular, dysregulation of lncRNAs has been discovered in many human cancers [12–14]. For example, HOX transcript antisense RNA (HOTAIR) is the first lncRNA [15] demonstrated to have a *trans*-transcriptional regulatory function, and it has been shown to have carcinogenic function in prostate cancer [16] and breast cancer [17, 18]. The expression of H19 is profoundly upregulated in hepatocellular carcinoma and bladder cancer and is associated with poor clinical prognosis [19, 20]. A previous report demonstrated that lncRNA UICLM (upregulated in colorectal cancer liver metastasis) expression in CRC promoted tumor growth and liver metastasis, and knockdown of lncRNA UICLM expression impaired cell sphere-forming ability and epithelial-mesenchymal transformation (EMT) [21, 22]. EMT is an important biological process of malignant tumor cells derived from epithelial cells to obtain the ability to migrate and invade [23]. In 2000, the zinc finger factor snail (Snail Homologue 1), hereinafter referred to as SNAI1, is a transcriptional suppressor that seems to control protein stability, nuclear localization and function by phosphorylation and zinc transport proteins. Its identification as a transcriptional inhibitor of CDH1 and an inducer of EMT is an important breakthrough and provides a new insight into the molecular mechanism of tumor invasion [24, 25]. SNAI1, a major regulator of EMT, was identified as an upstream of lncRNA WiNTRLINC1 [26]. Therefore, a better understanding of lncRNA and SNAI1 function in CRC progression will improve the understanding of molecular mechanisms and provide information for the diagnosis and treatment of CRC patients.

The lncRNA MAPKAPK5-AS1 (hereinafter referred to as MK5-AS1) is located on chromosome 12q24.12 and has been demonstrated to act as an oncogenic molecule [27]. In this study, we identified MK5-AS1 by screening publicly available expression profiling data from CRC and integrating bioinformatics analyses. MK5-AS1 was significantly upregulated in CRC tissues and correlated with clinical stages and metastasis of CRC patients. In addition, MK5-AS1 regulated cell proliferation and migration both in vitro and in vivo. LncRNAs, including MK5-AS1, have been shown to act as competitive endogenous RNAs (ceRNAs), which can regulate the expression of cancer-related genes by competing with miRNA response elements (MREs) [28, 29], thus affecting mRNA or other lncRNA transcripts. Additionally, we identified a novel regulatory pathway in which MK5-AS1 promoted metastasis via the let-7f-1-3p-SNAI1 axis in CRC. The other mechanism suggested that MK5-AS1 could recruit RBM4 and eIF4A1 to promote the translation of MK5 (MAPK activated protein kinase 5), while MK5 promoted the phosphorylation of c-Jun, and c-Jun activated the transcription of SNAI1 by directly binding to its promoter. These mechanisms demonstrated that non-protein-coding genes contributed to oncogenesis and elucidated the complex genetic rewiring that was driven by MK5-AS1 in CRC. All these results suggest that MAPKAPK5-AS1 could be a prognostic marker for CRC patients.

## Materials and methods

### Clinical specimens

Between January 2012 and October 2014, 172 pairs of CRC tumor tissues and corresponding adjacent normal tissues were collected from patients with CRC who underwent surgery at Xuzhou Medical University Affiliated Hospital (Xuzhou, China). Patients did not receive any radiotherapy or chemotherapy before surgery. Samples were pathologically confirmed and rapidly placed into liquid nitrogen after surgical operation until use. CRC patients were staged according to the TNM staging system (the 7th edition) of the American Joint Committee on Cancer. Details of the clinicopathological data are shown in Table 1. When the expression of MK5-AS1 was greater than the average, MK5-AS1 was considered to be high and vice versa. The study protocol was approved by the Research Ethics Committee of Xuzhou Medical University. Clinical specimens were obtained with the informed consent of patients.

**Table 1:** The clinic-pathological factors of CRC patients

Characteristics	Number of cases	MAPKAPK5-AS1 expression		*P* value^a^
		Low(*n* = 86)	High(*n* = 86)	
Age (year)				
≤ 60	91	46	45	0.500
> 60	81	40	41	
Gender				
Female	84	40	44	0.324
Male	88	46	37	
Tumor invasion depth				
T1–2	66	46	20	**<0.001**
T3–4	106	40	66	
Lymph node metastasis				
N0	101	66	35	**<0.001**
N1 + 2 + 3	71	20	51	
Distant metastasis				
M0	152	83	69	**0.001**
M1	20	3	17	
TNM stage				
I + II	77	53	24	**<0.001**
III + IV	95	33	62	

^a^Statistical significant results (in bold)

### Cell lines and culture

Human CRC cell lines (HCT116, SW620, SW480, DLD-1, HT-29) were purchased from the Cell Bank of the Chinese Academy of Science (Shanghai, China). The human normal colorectal epithelial cell line FHC was obtained from the American Type Culture Collection (Manassas, VA, USA). Cell lines were cultured in the appropriate medium supplemented with 10% fetal bovine serum (FBS; Gibco, NY, USA) and 1% antibiotic/antimycotic solution and maintained in an incubator at 37 °C with 5% CO_2_ in a humidified atmosphere. DLD-1 and HT-29 cells were maintained in RPMI-1640 medium (Gibco), FHC cells were cultured in Dulbecco’s modified Eagle medium (DMEM; Gibco), HCT116 cells were maintained in McCoy’s 5A medium (KeyGEN, Nanjing, China) and SW620 cells were maintained in L-15 medium (KeyGEN). To test the effects of the N-terminal phosphorylation of c-Jun, HCT116 and SW620 cells were pretreated with SR11302 (10 μM for 1 h) (APExBIO, USA) before transient transfection [30].

### Quantitative real-time PCR (qPCR)

According to the manufacturer’s instructions, total RNA from CRC tissues and cells was isolated and quantified using TRIzol reagent (Takara, China). LncRNAs, miRNAs and mRNAs were reverse transcribed in accordance with the manufacturer’s protocol by using PrimeScript RT Master Mix (Takara). The relative quantification of MK5-AS1 was carried out by the 2^-ΔΔCT^ method, and 18S rRNA was used as an internal control. The expression levels of let-7f-1-3p and miR-1284 were normalized to the levels of internal control U6 by the 2^-ΔΔCT^ method. The expression levels of MK5 and SNAI1 were normalized to the levels of the internal control GAPDH by the 2^-ΔΔCT^ method. The reactions were performed independently in triplicate. Quantitative PCR assays were carried out on ABI StepOne (Carlsbad, CA, USA). The primer sequences are listed in Table S1.

### Cell transfection

Negative control siRNA (si-NC) and four individual MK5-AS1 siRNAs (si-MK5-AS1 #1, #2, #3, #4), three individual MK5 siRNAs (si-MK5 #1, #2, #3), c-Jun siRNA, let-7f-1-3p mimics, and let-7f-1-3p inhibitor were purchased from Gene Pharma (Shanghai, China). The siRNA sequences are listed in Table S1. Cells were transfected with siRNA when at 30–50% confluence using siLentFect Lipid Reagent (Bio-Rad, CA, USA). MK5-AS1, MK5, and c-Jun were amplified from human cDNA as a template and were cloned into the pcDNA3.1(+) vector (Invitrogen, USA). The c-Jun-S63A sequence was thereafter generated using overlap extension PCR and cloned into pcDNA3.1(+). HCT116 and SW620 cells were grown to 90% confluence before being transiently transfected with plasmids using Lipofectamine 2000 (Invitrogen), according to the manufacturer’s protocol. At 24–48 h posttransfection, cells were harvested for qPCR or western blot analysis.

### Cell proliferation assay (CCK8 assay and colony formation assay)

A cell proliferation assay was performed with the Cell Counting Kit-8 (CCK8) assay (APExBIO). Equal numbers of HCT116 and SW620 cells 24 h after transfection with si-MK5-AS1, si-MK5, let-7f-1-3p inhibitor and let-7f-1-3p mimics were seeded into 96-well plates. CCK8 solution (10 μl per well) with 100 μl serum-free medium was added every 24 h, and the plates were incubated at 37 °C for 2 h. The optical density (OD) was then measured at 450 nm. For the colony formation assay, HCT116 and SW620 cells transfected similarly were plated in each well of a six-well plate and cultured in the appropriate medium containing 10% FBS for approximately 14 days, and the medium was replaced every 5 days. After 14 days, the colonies were fixed with methanol and stained with 0.1% crystal violet (Vicmed, China). The colony formation rate was determined by counting the number of stained colonies.

### Migration and invasion assays

The cell migration and invasion abilities were evaluated by a modified bicameral culture system with a pore size of 8 μm. Transwell inserts (Corning Incorporated, USA) with or without Matrigel (BD Biosciences, USA) coating were used to perform the invasion or migration assays, respectively. The transfected cells were seeded into transwell inserts. After culture, cells were fixed with 4% paraformaldehyde solution and stained with 0.1% crystal violet. Then, we used an Olympus microscope to obtain images at a magnification of × 100 and then used ImageJ software to calculate the number of cells penetrating the pores. All experiments were carried out three times.

### Immunoblotting, coimmunoprecipitation (co-IP) and antibodies

Cells were harvested with RIPA lysis buffer (Beyotime, China) supplemented with PMSF, phosphatase inhibitor cocktail and protease inhibitor cocktail (Sigma Aldrich, MO, USA). Lysates were cleared by centrifugation at 13,000 g for 15 min at 4 °C. Cell protein lysates were quantified and separated by electrophoresis on SDS-polyacrylamide gel electrophoresis, transferred to a nitrocellulose filter membrane, blocked with 5% skim milk (BD Biosciences) in Tris-buffered saline with 0.05% Tween-20, and probed with specific antibodies. The signals were detected using Chemistar™ High-sig ECL Western Blot Substrate (Tanon, Shanghai, China). Similarly, human tissues were ground and then prepared in RIPA buffer as mentioned before. For co-IP, cell lysates (1000 μg) containing a cocktail of protease/phosphatase inhibitors were rotated overnight for immunoprecipitation with anti-c-Jun IgG, anti-MK5 IgG, anti-RBM4 IgG, anti-eIF4A1 IgG and rabbit IgG (Beyotime, China). Then, 30 μl of Protein A/G agarose beads (Santa Cruz Biotechnology, USA) was added to the cell lysates and incubated for 4 h at 4 °C. Beads were washed with lysis buffer three times. The immunoprecipitation complexes were analyzed by western blot. Antibodies against the following proteins were used: MK5 (1:1000; Santa Cruz Biotechnology); c-Jun (1:200; Santa Cruz Biotechnology); p-c-Jun (1:5000; Abcam, USA); N-cadherin (1:2000; Abcam); E-cadherin, vimentin, SNAI1, SNAI2, and RBM4 (1:1000; Proteintech, Wuhan, China); eIF4A1 (1:1000, Cell Signaling Technology, USA); GAPDH (1:1000; ABM, Canada); goat anti-rabbit HRP, and goat anti-mouse HRP (1:10,000, Vicmed).

### RNA-Fluorescence in Situ Hybridization (RNA-FISH)

HCT116 cells were washed twice with phosphate buffered saline (PBS) and fixed in 4% formaldehyde for 15 min. The fixed cells were permeabilized with Triton X-100 and dehydrated by an ascending series of ethanol concentrations. The cells were then incubated with 50 nmol probe labeled with CY3 at the 5′ end in hybridization buffer at 73 °C for 5 min. Cells were hybridized at 37 °C for 14 h, washed and dehydrated. After adding DAPI working solution, the cells were scanned and imaged. RNA FISH probes were designed and synthesized by Sangon Biotech (Shanghai, China). The probe sequences are listed in Table S1.

### RNA pull-down assay

Biotin-labeled RNAs were transcribed with T7 RNA polymerase (Takara) and biotin labeling mix (Roche, USA). The product was treated with RNase-free DNase I and purified with the RNeasy Mini kit (Qiagen, MD, USA). We used the Pierce Magnetic RNA-Protein Pull-Down Kit (Thermo Fisher, USA) according to the manufacturer’s instructions. The biotin-labeled RNAs (1 μg) were first folded in RNA structure buffers (20 mM Tris-HCl [pH 7.0], 0.2 M KCl and 20 mM MgCl_2_) for 20 min and then coincubated with HCT116 cell lysates and 100 U/ml RNase inhibitor at 4 °C for 12 h. After incubation, the RNA/protein complex was captured by magnetic streptavidin-coupled beads, washed twice in washing buffer and eluted in elution buffer. The eluted lncRNA-interacting proteins were separated by SDS–PAGE and standard immunoblotting.

### RNA Immunoprecipitation (RIP) assay

HCT116 cells lysates were incubated with anti-RBM4 IgG or anti-eIF4A1 IgG at 4 °C for 12 h. The immune complex was conjugated with 30 μl Protein A/G beads. After 3–6 h, the beads were washed and then directly resuspended in TRIzol reagent and subjected to RNA isolation. Finally, specific primers were used for qPCR analysis of immunoprecipitated RNA to confirm the presence of MK5-AS1.

### Dual luciferase reporter assay

The sequences of human MK5-AS1 and SNAI1 3’UTR were cloned into pGL3-basic (Promega, USA) at the *XbaI* site. Similarly, the SNAI1 promoter was cloned into pGL3-basic between the *KpnI* and *XhoI* sites. All mutant SNAI1 promoter and 3’UTR sequences were amplified by overlap extension PCR. HCT116 cells seeded in a 24-well plate were cotransfected with expression plasmids and RNA oligos using Lipofectamine 2000. The relative activity of firefly luciferase was assessed by the Dual-Luciferase Reporter Assay System (Promega) after 48 h of transfection and normalized to that of Renilla luciferase. All experiments were repeated in triplicate.

### Chromatin Immunoprecipitation (ChIP) assays

The ChIP assay was performed with an EZ-ChIP kit (Millipore, USA) according to the manufacturer’s protocol. Whether or not HCT116 and SW620 cells were transfected with c-Jun, they were lysed by SDS lysis solution. The DNA samples were reduced to 500–1000-bp fragments by ultrasound. DNA fragments were coincubated with anti-c-Jun IgG, rabbit IgG or anti-RNA pol II IgG at 4 °C for 12 h. The immune complex was conjugated with 40 μl Protein A/G beads. After 1–2 h, the protein/DNA complexes were washed and then eluted in an elution buffer containing 1% SDS and 0.1 M NaHCO_3_, and the DNA was purified by a DNA cleanup spin column. The presence of specific DNA sequences in the eluted sample was measured by PCR. The ChIP primers are listed in Table S1.

### Lentiviral transduction and tumor xenograft experiments

HCT116 cells labeled with luciferase were infected with lentiviruses expressing GFP and carrying sh-negative control, sh-MAPKAPK5-AS1, or sh-MK5 by 8 mg/ml Polybrene (Gene Pharma). The shRNA sequences are listed in Table S1. All animal experiments were approved by the Committee on the Ethics of Animal Experiments of the Xuzho u Medical University. All animal studies complied with the National Institutes of Health Guide for the Care and Use of Laboratory Animals. BALB/c female nude mice were provided by HFK Bioscience (Beijing, China) and randomized into four groups (*n* = 7 for each). A total of 3 × 10^6^ Ctrl-HCT116 cells, sh-MK5-AS1 HCT116 cells and sh-MK5 HCT116 cells suspended in 150 μl PBS were injected into mice through the tail vein. The metastatic tumor numbers were measured every 3 days with a whole-body fluorescent imaging system. After 2 months, mice in all four groups were sacrificed, and pulmonary tumors were isolated for imaging, statistical analysis and hematoxylin and eosin (HE) staining.

### H&E and immunohistochemical staining

Paraffin-embedded lung tissues were sectioned at 5 μm thick, then stained with hematoxylin and eosin (H&E). Pulmonary tissues were cut into 4 μm sections, then deparaffinized and rehydrated. For retrieving antigen, the slides were heated at 95 °C in 0.01 M citrate buffer (pH = 6.0), and 3% hydrogen peroxide was used to quench peroxidase activity for 20 min. The sections were treated with normal goat serum, followed by incubation overnight with anti-Vimentin antibody (1:400 dilution; Proteintech) at 4 °C. After being rinsed with PBS, the sections were incubated with goat anti rabbit IgG for 1 h and stained with 3, 3′-diaminobenzidine (DAB; Zhongshan biotech, Beijing, China). After hematoxylin counterstain was completed, all the sections were dehydrated and sealed. We used PBS instead of prime antibody as a negative control. All images were recorded by Olympus BX-51 light microscope.

### Statistical analysis

Statistical analysis was performed by SPSS 17.0 software (SPSS, USA), and images were acquired with GraphPad Prism 5 software (La Jolla, USA). The significance of the differences between the groups was evaluated by a paired two-tailed Student’s t-test or χ2 test. The correlation analysis between MK5-AS1, let-7f-1-3p and MK5 was evaluated by Spearman’s test. The Kaplan-Meier method and log-rank test were used to evaluate the overall survival (OS). Data represent the mean ± standard deviation (SD). Differences were considered statistically significant when *P* < 0.05 (* *P* < 0.05, ** *P* < 0.01, *** *P* < 0.001).

## Results

### MK5-AS1 was upregulated in CRC

**Fig. 1:**
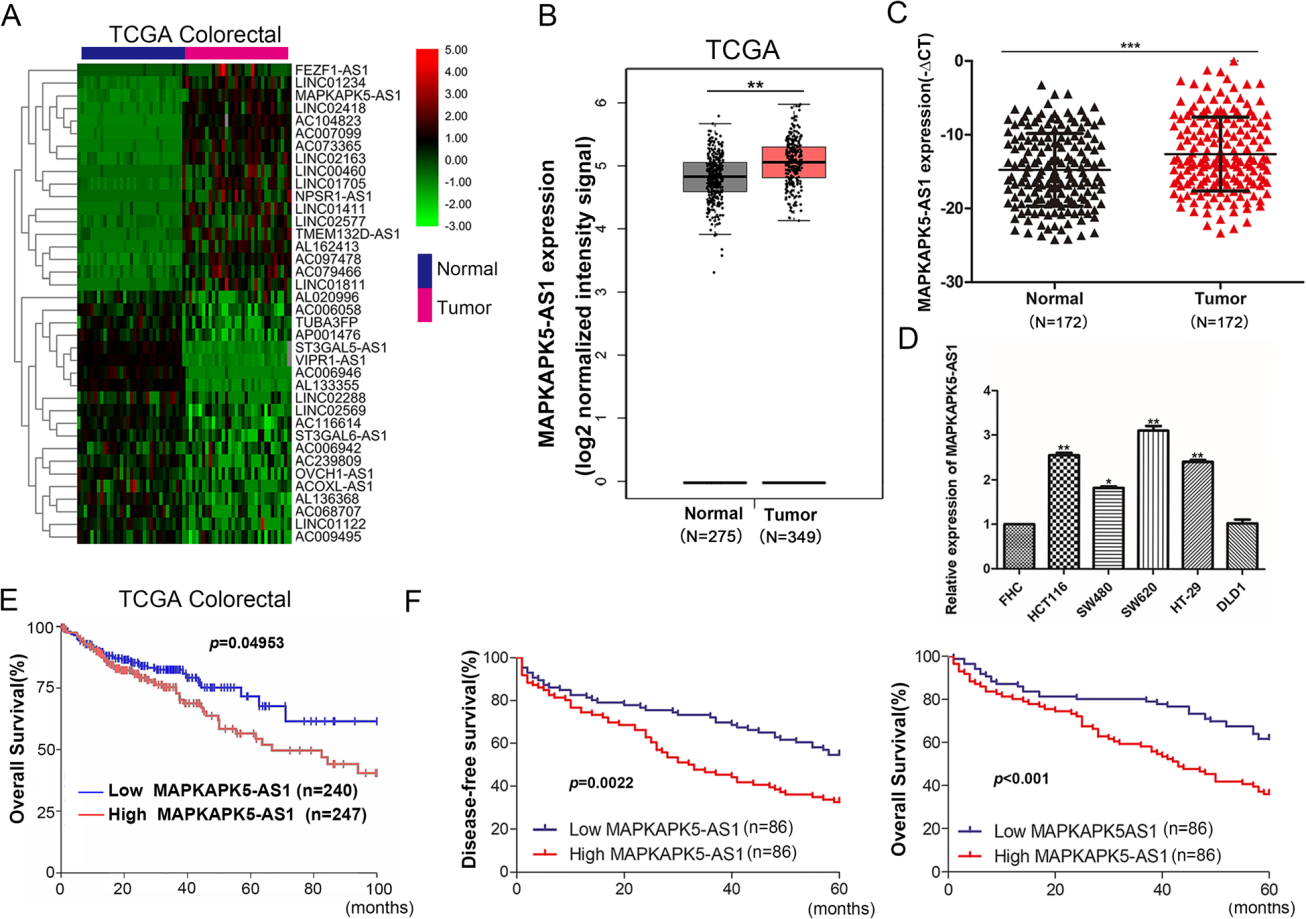
MK5-AS1 was upregulated in CRC tissues. **a.** A Heatmap of RNA-Seq analysis of differentially expressed lncRNAs in CRC and corresponding normal tissues generated from RNA sequencing data from the TCGA database. Red in the heatmap denotes upregulation, green denotes downregulation. **b.** Expression of MK5-AS1 in the TCGA CRC cohort. **c.** qPCR was utilized to analyze the MK5-AS1 expression in 172 pairs of CRC tissues and corresponding adjacent non-tumoral tissues. **d.** Expression of MK5-AS1 was detected by qPCR in the normal colorectal epithelium cell line (FHC) and CRC cell lines. **e.** Kaplan-Meier overall survival was analyzed according to MK5-AS1 expression in TCGA cohort. **f.** Kaplan-Meier overall survival and relapse-free survival curves according to MK5-AS1 expression in our cohort. *P < 0.05, **P < 0.01 and ***P < 0.001

To discover the lncRNAs that was involved in CRC, we first analyzed the lncRNA expression profiles of CRC in TCGA colorectal database. We identified a series of abnormally expressed lncRNAs in CRC and found that MAPKAPK5-AS1 was significantly upregulated (Fig. 1a). Analysis of the TCGA dataset confirmed that MK5-AS1 was upregulated in CRC tissues compared with normal tissues (Fig. 1b). Therefore, we speculated that it may play an important role in the development and homeostasis of colorectal tissue. To investigate MK5-AS1 expression in CRC, qPCR was performed in 172 pairs of matched colorectal tissues and corresponding normal tissues. The results showed that the levels of MK5-AS1 were upregulated in CRC tissues (Fig. 1c). In addition, we measured the levels of MK5-AS1 in CRC cell lines (HCT116, SW480, SW620, HT-29, DLD-1) and the normal colorectal epithelial cell line FHC by qPCR. The results showed that MK5-AS1 expression was increased in CRC cell lines compared with the normal cell line (Fig. 1d). Further analysis of clinical data revealed a correlation between the expression of MK5-AS1 and the clinicopathology of CRC patients. Correlation analysis demonstrated that high levels of MK5-AS1 were associated with tumor invasion depth (*P* < 0.001), lymph node metastasis (*P* < 0.001), Distant metastasis (*P* = 0.001) and TNM stages (*P* < 0.001). However, there was no significant relationship between MK5-AS1 expression and other factors, including age (*P* = 0.5) and gender (*P* = 0.324) (Table 1). To further determine whether the increase in MK5-AS1 staining in patients with CRC was associated with poor prognosis, Kaplan-Meier analysis showed that high levels of MK5-AS1 in the TCGA cohort were associated with decreased overall survival (OS), while high levels of MK5-AS1 in our CRC cohort were associated with overall survival (OS) and relapse-free survival (RFS) (Fig. 1e and f). Cox univariate regression analysis showed that MK5-AS1 expression was an independent prognostic factor in patients with CRC (Table 2). Therefore, these results showed that it may play an important role in the progression and homeostasis of colorectal cancer.

**Table 2:** Cox univariate regression analysis of the risk factors for death in CRC patients

Variables^a^	Overall survival		Relapse-free survival	
	HR (95% CI)	*P*	HR (95% CI)	*P*
MK5-AS1	0.476 (0.309–0.734)	**0.01**	0.538 (0.358–0.808)	**0.003**
Age (year)	0.777 (0.501–1.203)	0.258	0.667 (0.439–1.015)	0.059
Gender	0.854 (0.561–1.301)	0.463	0.885 (0.581–1.348)	0.57
Tumor invasion depth	0.463 (0.312–0.688)	**< 0.001**	0.48 (0.323–0.713)	**< 0.001**
Lymph node metastasis	0.319 (0.19–0.536)	**< 0.001**	0.355 (0.211–0.598)	**< 0.001**
Distant metastasis	0.112 (0.033–0.383)	**< 0.001**	0.228 (0.075–0.694)	**0.009**
TNM stage	0.412 (0.27–0.63)	**< 0.001**	0.426 (0.279–0.653)	**< 0.001**

^a^MK5-AS1: low vs high; age: ≤60 vs > 60; gender: male vs female; Tumor invasion depth: T1–T2 vs T3–T4; Lymph node metastasis: N0 vs N1, N2, N3; TNM stage was ranked as I–II vs III–IV

### MK5-AS1 promoted the proliferation, migration and invasion of CRC

Because HCT116 and SW620 cells had the highest expression of MK5-AS1 among the CRC cell lines, they were selected for further study in subsequent experiments. To assess the biological functions of MK5-AS1 in cell proliferation, migration and invasion of CRC, transient transfection of MK5-AS1-expressing cell lines was performed, wherein pcDNA3.1 MK5-AS1 and siMK5-AS1-siRNA (si-MK5-AS1 #1, si-MK5-AS1 #2, si-MK5-AS1 #3, si-MK5-AS1 #4) were used for overexpression and knockdown functional studies, respectively. The levels of MK5-AS1 in the transient transfection cell lines were detected by qPCR to demonstrate the effectiveness of transfections. Next, si-MK5-AS1 #1 and si-MK5-AS1 #2 were selected for further experiments on the basis of their more effective inhibition (Fig. S1A and S1B). The growth curve of the CCK8 proliferation assay showed that MK5-AS1 knockdown clearly inhibited the growth of HCT116 and SW620 cells, while MK5-AS1 overexpression promoted the proliferation of HCT116 and SW620 cells (Fig. S1C). The same results were obtained by colony formation assay. Colony formation experiments showed that MK5-AS1 decreased the number and size of the colonies in HCT116 and SW620 cells and that MK5-AS1 overexpression was upregulated (Fig. S1D). These results demonstrate that MK5-AS1 can promote the proliferation of CRC cells.

**Fig. 2:**
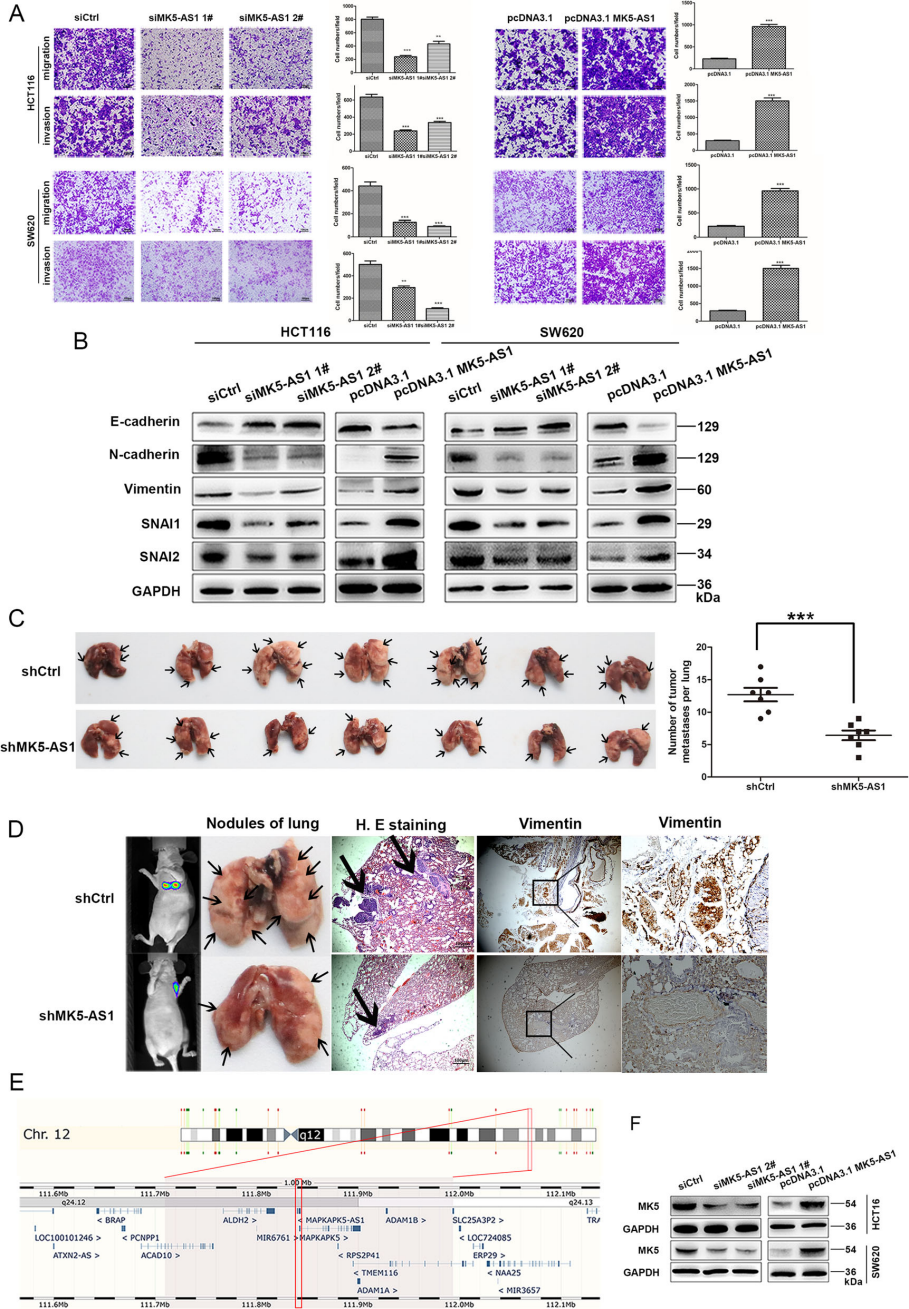
MK5-AS1 regulated CRC invasion and migration in vitro and *vivo*. **a.** Transwell assays were used to determine the invasion and migration abilities of HCT116 and SW620 cells after MK5-AS1 overexpression and knockdown. Scale bar, 100 μm for A. **b.** Immunoblotting of EMT-related markers after transfection in CRC cells. **c.** Nude mice were injected with HCT116 cell after MK5-AS1 knockdown into tail vein. The number of metastatic nodules of lung was shown and counted. The black arrow marked metastatic nodules. **d.** Tumor progression was monitored using a small animal imaging system, HE-stained lung sections (Scale bar, 100 μm) and antibody vimentin of metastatic nodules were shown (× 400 magnification). The black arrow marked metastatic nodules. **e.** The Ensembl Genome browser (http://asia.ensembl.org/) showed that MK5 was the nearby gene of MK5-AS1. **f.** Immunoblotting was used to investigate the level of MK5 of HCT116 and SW620 cells after intervening MK5-AS1, respectively. The data represented the mean ± SD from three independent experiments. *P < 0.05, **P < 0.01 and ***P < 0.001

Next, transwell assays revealed that knockdown of MK5-AS1 expression clearly suppressed migration and invasion compared with the control transfection. As expected, overexpression of MK5-AS1 promoted cell migration and invasion (Fig. 2a). Local tumor invasion is the first step in the cascade of tumor metastasis, which requires profound changes in the adhesion and migration of tumor cells, which is reminiscent of the developing EMT. To further verify that MK5-AS1 promoted the migration and invasion of CRC, we detected the levels of EMT-related markers. The results showed that MK5-AS1 knockdown markedly increased epithelial marker E-cadherin expression and decreased the expression of the mesenchymal markers N-cadherin, Vimentin, SNAI1 and SNAI2. Conversely, MK5-AS1 overexpression attenuated the levels of E-cadherin and boosted the levels of N-cadherin, Vimentin, SNAI1 and SNAI2. The change in SNAI1 levels was especially obvious (Fig. 2b). To determine whether MK5-AS1 affected tumor metastasis in vivo, HCT116 cells stably transfected with control vector or sh-MK5-AS1 were injected into the caudal vein of nude mice. According to previous studies, vimentin was used to detect lung metastasis in nude mice [31]. The results demonstrated that there were less metastatic foci in the lungs of nude mice in the MK5-AS1 knockdown groups than in the empty vector group, and micrometastases detected by hematoxylin and eosin (H&E) and anti-vimentin staining (Fig. 2c and d). Collectively, these results revealed that MK5-AS1 is an oncogene that is associated with proliferation, migration and invasion.

### MK5 promoted CRC proliferation, migration, and invasion

**Fig. 3:**
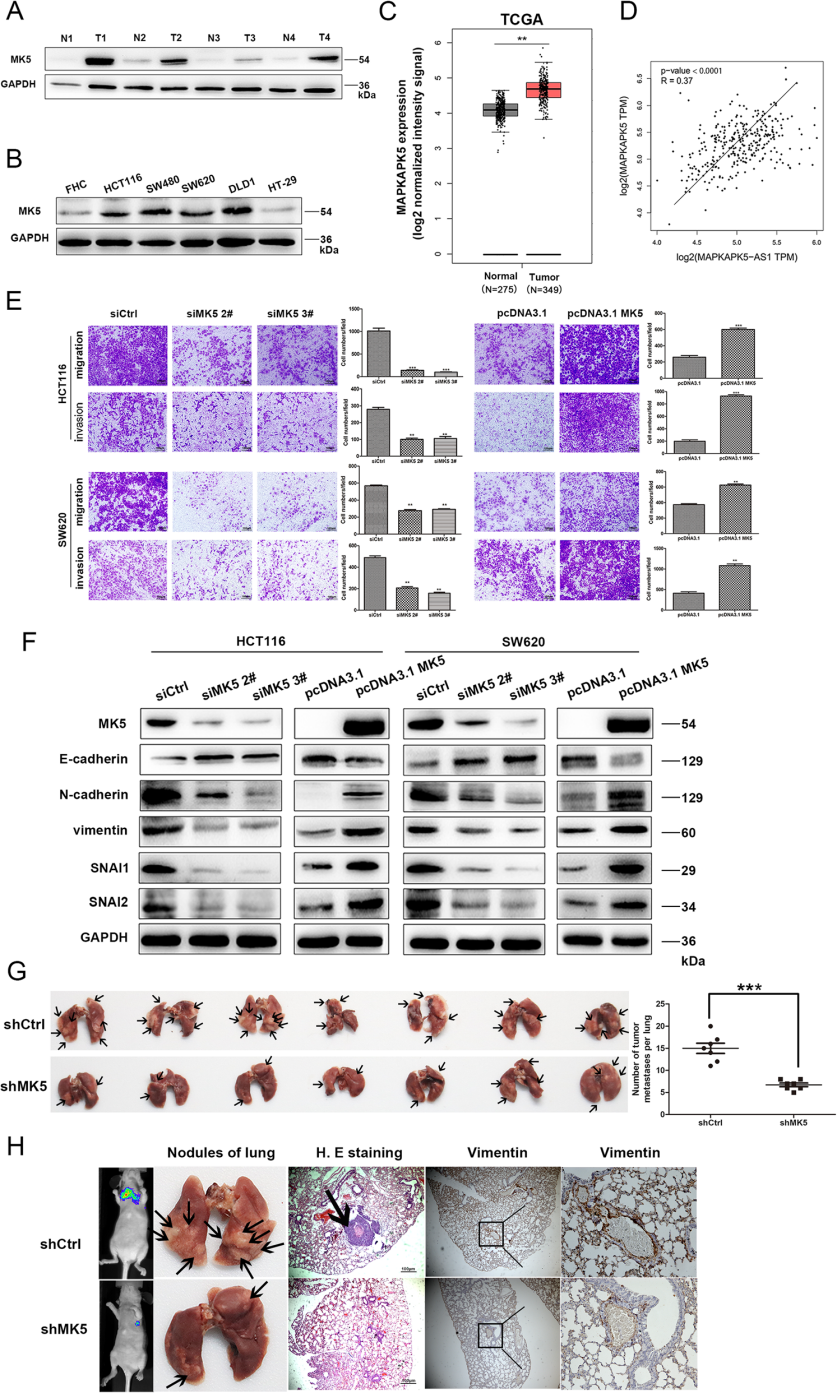
MK5 promoted CRC cells invasion and migration in vitro and *vivo*. **a.** Immunoblotting of MK5 expression in 4 pairs of human CRC tissues (T) and adjacent non-tumor tissues (N). **b.** Expression of MK5 in the normal colorectal epithelium cell line (FHC) and CRC cells by immunoblotting. **c.** Statistical analysis of MK5 expression in TCGA database. **d.** Correlation analysis of the expression of MK5-AS1 and MK5 in GEPIA. **e.** Transwell assays were used to determine the changes in invasion and migration abilities of HCT116 and SW620 cells after transfection. Scale bar, 100 μm for E. **f.** Immunoblotting analysis of EMT-related markers after transfection in CRC cells, respectively. **g.** The number of metastatic pulmonary nodules was shown and counted. The black arrow marked metastatic nodules. **h.** After HCT116 cells with knockdown MK5 were injected into the tail vein of nude mice, In vivo fluorescence imaging, the gross lesion in lung tissues and H. E staining and antibody vimentin of metastatic nodules in the lungs were observed (× 400 magnification). Scale bar, 100 μm for H.E. The black arrow marked metastatic nodules. The data represented the mean ± SD from three independent experiments. *P < 0.05, **P < 0.01 and ***P < 0.001

Previous reports [7] have shown that lncRNAs can be widely divided into those that function in *cis*, thus affecting the expression and/or chromatin state of nearby genes, and those that perform a series of functions in a *trans* manner throughout the cell. The biological behavior induced by MK5-AS1 was likely to occur in whole or in part through the effect of the MK5 gene, which has partially overlapping sequences with MK5-AS1 (Fig. 2e). In this context, our data showed that there were changes in the expression of MK5 protein and mRNA after knockdown or overexpression of MK5-AS1 (Fig. 2f and Fig. S1E). Additionally, we used western blot to detect the levels of MK5 in 4 pairs of matched colorectal tissues and corresponding normal tissues and in CRC cell lines (HCT116, SW480, SW620, HT-29, DLD-1) and the normal colorectal epithelial cells FHC. The results showed that the expression of MK5 in CRC tissues and cells was considerably increased compared to that in adjacent nontumor tissues and normal cells (Fig. 3a and b). Furthermore, analysis of the TCGA dataset and western blot demonstrated that MK5 expression was significantly upregulated in CRC tissues (Fig. 3c and Fig. S2). Kaplan–Meier analysis of OS showed that our CRC patients, but not the CRC patients of the TCGA cohorts, with high MK5 expression had a much worse prognosis than those with low MK5 levels (Fig. S3A). Further analysis showed that MK5 was positively correlated with MK5-AS1. Similarly, the correlation between MK5 and MK5-AS1 was also congruent with the publicly available GEPIA data (Fig. 3d).

Furthermore, to assess the biological functions of MK5 in cell proliferation, migration and invasion of CRC, we knocked down or overexpressed MK5 in HCT116 and SW620 cells by transfecting with si-MK5 (si-MK5 #1, siMK5 #2, siMK5 #3) or with an overexpression plasmid (pcDNA3.1-MK5) (Fig. S3B). Then, si-MK5 #2 and si-MK5 #3 were selected for further experiments on the basis of their more effective inhibition. Additionally, we examined the function of MK5 on CRC cells using CCK8 and colony formation assays. The results of the CCK8 assay demonstrated that MK5 silencing downregulated cell proliferation in HCT116 and SW620 cells compared with the control cells, while MK5 upregulated cell growth (Fig. S3C). Analogously, the colony formation assay of MK5 knockdown and overexpression cells revealed that MK5 knockdown cells formed fewer colonies than control cells, while MK5 overexpression cells formed more colonies (Fig. S3D). In summary, these results confirmed the promoting effect of MK5 on cell growth. Moreover, transwell assays showed that MK5 knockdown strikingly decreased cell migration and invasion, while MK5 overexpression increased cell migration and invasion (Fig. 3e). The initiation of EMT is the driving force for tumor invasion and metastasis. MK5 silencing upregulated the protein expression of the epithelial marker E-cadherin and downregulated the mesenchymal markers N-cadherin, Vimentin, SNAI1 and SNAI2, and the opposite was observed upon MK5 overexpression (Fig. 3f). We evaluated the effect of MK5 on tumor metastasis in vivo. By calculating the number of pulmonary nodules after intravenous injection of tumor cells in nude mice, we found that sh-MK5 markedly decreased the pulmonary homing potential of HCT116 cells (Fig. 3d). Micrometastases detected by H&E and anti-vimentin staining (Fig. 3h). Overall, these results suggested that sh-MK5 reduced lung colonization of tumors.

### MK5-AS1 promoted translation initiation by binding directly with RBM4

**Fig. 4:**
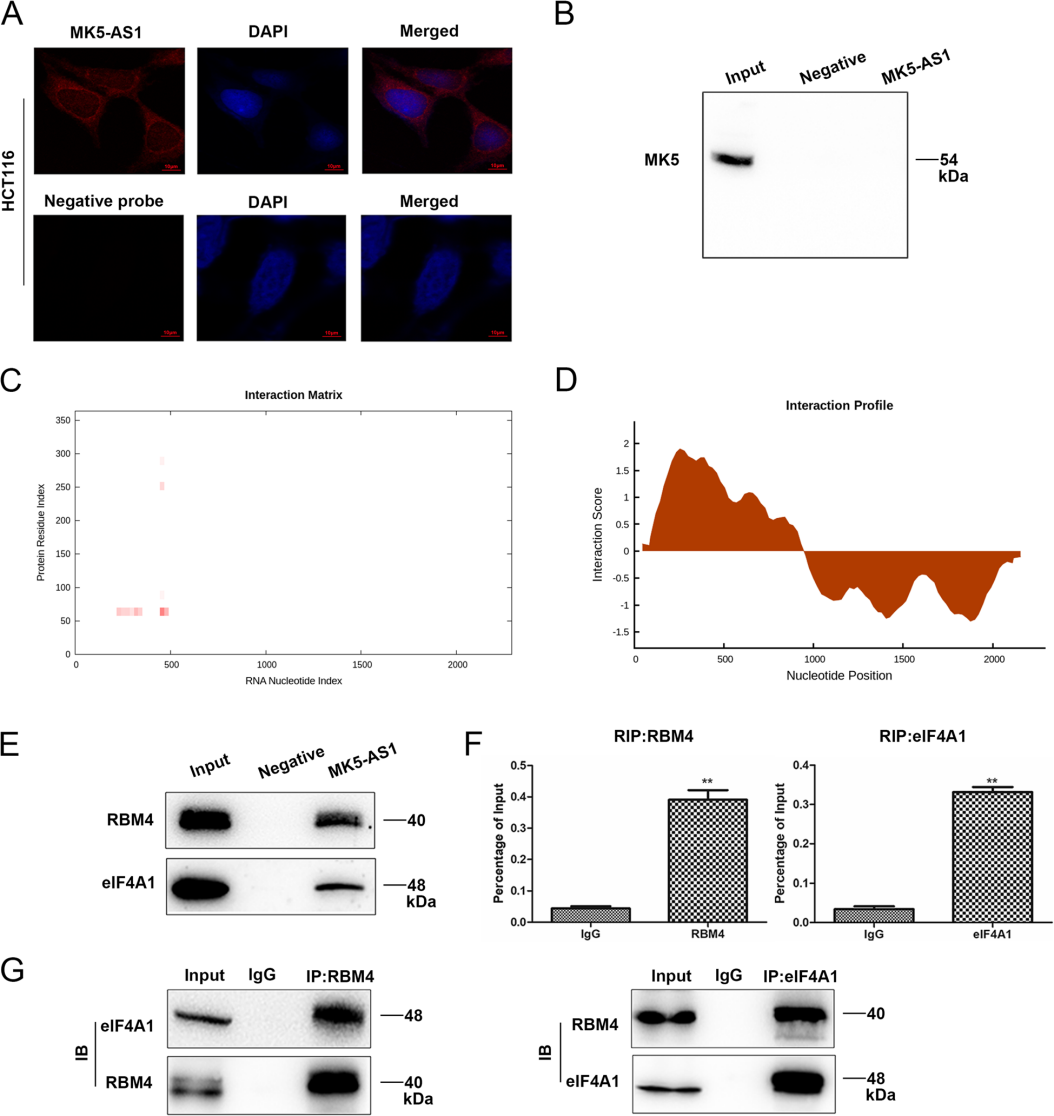
MK5-AS1 recruited RBM4/eIF4A1. **a.** RNA FISH analysis of the location of MK5-AS1 (red) in the cytoplasm of HCT116 cells. Original magnifications, × 400 for A. **b.** RNA pull-down assay showed that MK5-AS1 could not retrieve MK5 from HCT116 cells lysates. **c, d**. Analysis of the interaction propensities between MK5-AS1 and RBM4 by public databases catRAPID. **e.** RNA pull-down assays followed by immunoblotting showed that MK5-AS1 bound RBM4 and eIF4A1 in HCT116 cells. **f.** RIP experiments for RBM4 and eIF4A1 were performed and the coprecipitated RNA was subjected to qPCR for MK5-AS1. **g.** Coimmunoprecipitation was used to identify interaction between RBM4 and eIF4A1 in HCT116 cells. Data were shown as mean ± SD for three independent experiments. *P < 0.05, **P < 0.01 and ***P < 0.001

To explore the molecular mechanism of MK5-AS1 promoting the invasion and migration of CRC cells, we first analyzed the distribution of MK5-AS1 in CRC cells by using RNA-FISH. The results confirmed that MK5-AS1 was localized predominantly in the cell cytoplasm rather than the nucleus (Fig. 4a), indicating that MK5-AS1 might exert a major regulatory effect at the posttranscriptional level. To explore the mechanism between MK5-AS1 and MK5, we examined whether MK5-AS1 directly interacted with MK5 to facilitate MK5 expression. We performed an RNA pull-down experiment by western blot with anti-MK5 antibodies and could not retrieve MK5 from HCT116 cell extracts (Fig. 4b). Therefore, we speculated that MK5-AS1 might regulate MK5 expression posttranscriptionally through other pathways. We incubated the transcribed MK5-AS1 with beads coupled to protein lysate of HCT116 cells to purify the MK5-AS1 RNA-protein complex. Before the western blot experiment, we utilized the public bioinformatics databases catRAPID and RBPDB and predicted that RBM4 might interact with MK5-AS1 (Fig. 4c and d). The presence of RBM4 (RNA-binding motif 4), an RNA binding protein (RBP), was subsequently verified by western blot. Previous reports have demonstrated that RBM4 can interact with eIF4A1 [32] and participate in the initiation of translation. The RNA pull-down assay showed that MK5-AS1 RNA but not the negative control RNA specifically pulled down eIF4A1 from the HCT116 cell lysate (Fig. 4e). In addition, the relationship between RBM4, eIF4A1 and MK5-AS1 was confirmed in HCT116 cells by RIP assay (Fig. 4f). Based on these results, we hypothesized that RBM4 could recruit eIF4A1 to promote translation initiation. To confirm this mechanism, we conducted a co-IP experiment, and the results showed that RBM4 could physically interact with eIF4A1 (Fig. 4g).

### MK5 promoted SNAI1 expression by phosphorylating c-Jun

**Fig. 5:**
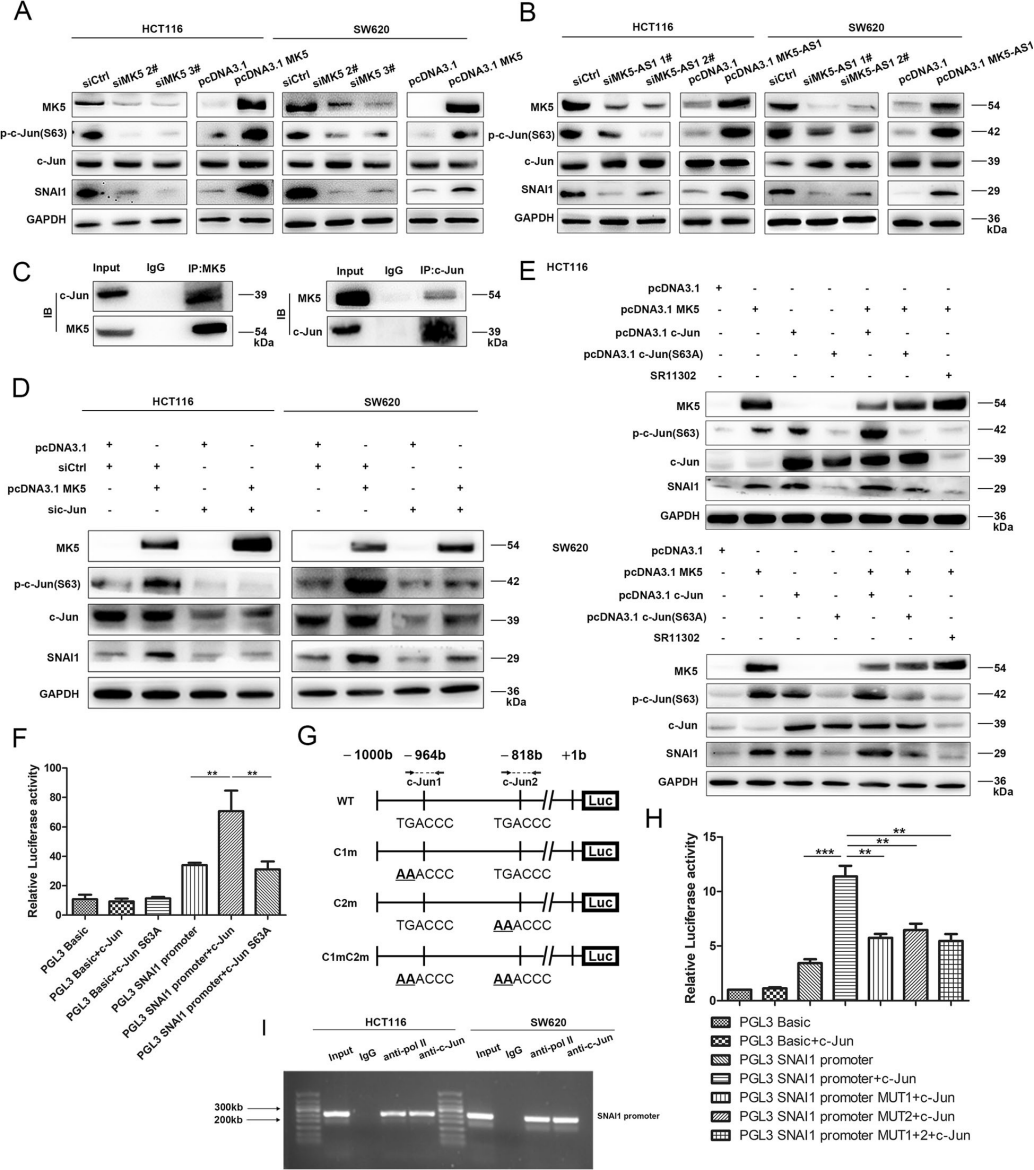
MK5 upregulated SNAI1 expression by phosphorylating c-Jun. **a.** Immunoblotting was confirmed the protein levels of c-Jun, p-c-Jun(S63) and SNAI1 after MK5 overexpression and silencing in HCT116 and SW620 cells. **b.** Immunoblotting analysis of MK5, c-Jun, p-c-Jun(S63) and SNAI1 after MK5-AS1 overexpression and knockdown in HCT116 and SW620 cells. **c.** Coimmunoprecipitation was used to identify interaction between MK5 and c-Jun in HCT116 cells. **d.** After cotransfection with pcDNA3.1 MK5, si-c-Jun or control siRNA, the proteins levels of MK5, c-Jun, p-c-Jun (S63) and SNAI1 were determined by immunoblotting. **e.** Immunoblotting of MK5, c-Jun, p-c-Jun(S63) and SNAI1 proteins levels from pcDNA3.1 MK5, pcDNA3.1 c-Jun, pcDNA3.1 c-Jun (S63A) transfection samples treated with DMSO or c-Jun phosphorylation inhibitor SR11302 in HCT116 and SW620 cells. **f.** Dual luciferase reporter plasmids containing SNAI1 promoter or pGL3 basic were co-transfected into HCT116 cells with c-Jun or mutant c-Jun (S63A) in parallel. **g.** Dual luciferase reporter plasmids of SNAI1 promoter were designed to contain c-Jun binding sequences or not. **h.** Dual luciferase reporter plasmids containing SNAI1 promoter, SNAI1 promoter MUT or pGL3 basic were cotransfected into HCT116 cells with c-Jun plasmid in parallel. **i.** Identification of the c-Jun binding sequences in SNAI1 promoters by ChIP-PCR. Data were shown as mean ± SD for three independent experiments. *P < 0.05, **P < 0.01 and ***P < 0.001

We found that intervening with MK5 expression in CRC cells could obviously affect the expression of SNAI1. Western blot analysis showed that MK5 could promote the phosphorylation of c-Jun S63 site and the expression of SNAI1 (Fig. 5a). Furthermore, after overexpression or knockdown of MK5-AS1, we observed phosphorylated c-Jun coincident with SNAI1 expression (Fig. 5b). Based on these results, we hypothesized that MK5-AS1 increased SNAI1 expression by promoting MK5 expression, which successively contributed to the phosphorylation of the transcription factor c-Jun. We carried out co-IP experiments, and the results showed that MK5 could physically interact with c-Jun (Fig. 5c). Overexpression of MK5 clearly increased the p-c-Jun and SNAI1 levels in HCT116 and SW620 cells, which could be reversed by transfection with si-c-Jun (Fig. 5d). Notably, co-overexpression of MK5 and c-Jun resulted in much greater expression of p-c-Jun and SNAI1 compared with each individual overexpression alone. In addition, mutation of the c-Jun S63 site (c-Jun S63A) or treatment with SR11302 (inhibitor of c-Jun phosphorylation) could not promote the expression of p-c-Jun and SNAI1 (Fig. 5e). These results indicated that MK5 promoted the expression of SNAI1 by phosphorylating c-Jun. In addition, compared with cotransfection with c-Jun and mutant SNAI1 promoter or cotransfection with c-Jun S63A and SNAI1 promoter, cotransfection of c-Jun and saturated SNAI1 promoter resulted in obvious activation of luciferase activity in HCT116 cells (Fig. 5f-h). The lysates containing DNA fragments of HCT116 and SW620 cells were incubated with anti-c-Jun antibody to purify the DNA-protein complexes. Then, it was verified by PCR that c-Jun could bind to the promoter of SNAI1 (Fig. 5i, Fig. S4A and S4B). According to the analysis, we found that MK5 promoted the phosphorylation of c-Jun, and c-Jun activated the transcription of SNAI1 by directly binding to its promoter.

### MK5-AS1 acted as a ceRNA to regulate SNAI1 expression by sponging let-7f-1-3p

**Fig. 6:**
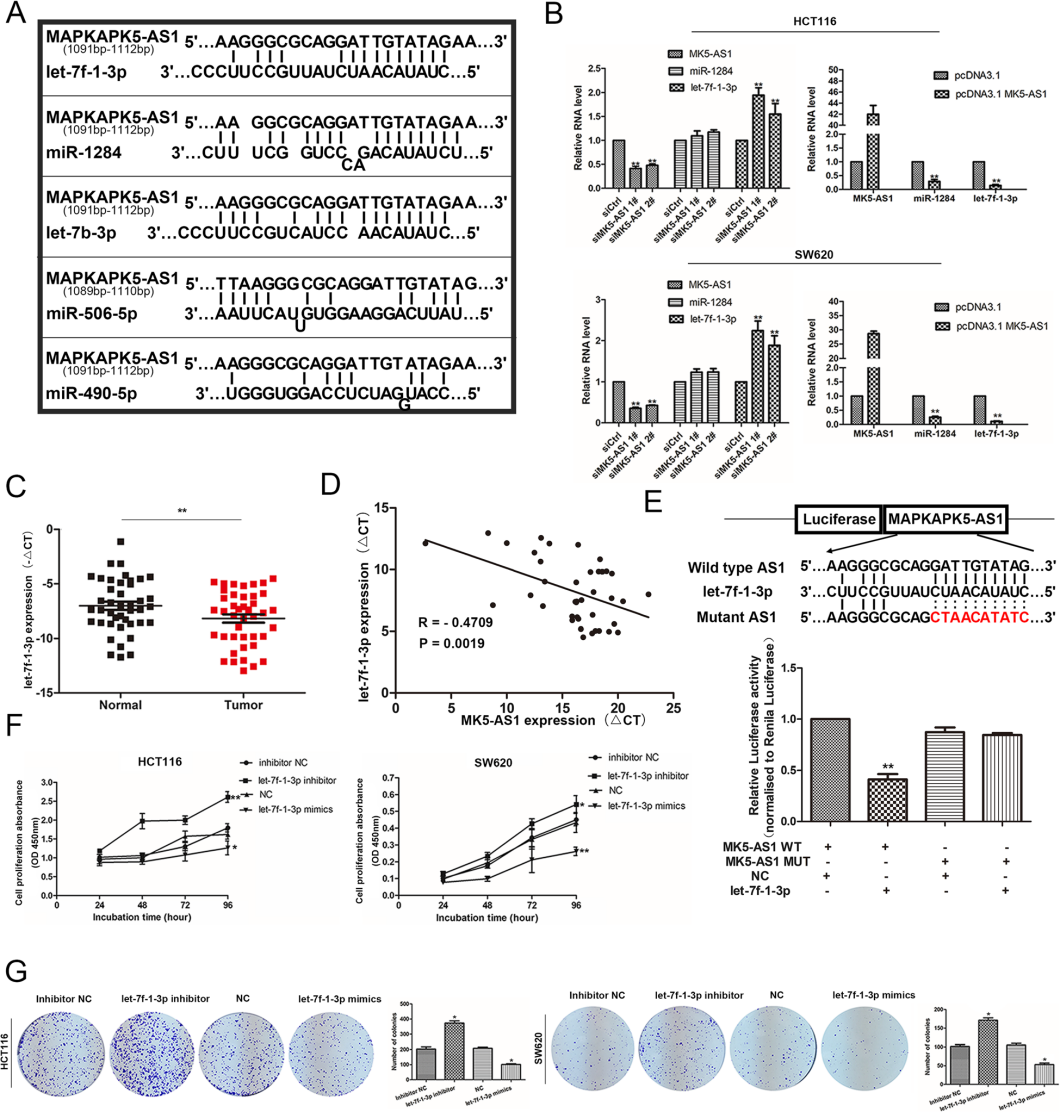
MK5-AS1 negatively regulated let-7f-1-3p expression. **a.** The possible binding sites among MK5-AS1 and microRNAs were predicted by DIANA Tools and miRcode. **b.** The expressions of miR-1284 and let-7f-1-3p were detected by qPCR in HCT116 and SW620 cells after intervening MK5-AS1, respectively. **c.** Let-7f-1-3p was downregulated in 42 pairs of CRC tissues compared with normal tissues. **d.** Correlation analysis of the expression of MK5-AS1 and let-7f-1-3p in 42 pairs of CRC tissues. **e.** Upper panel, the potential binding sites between MK5-AS1 and let-7f-1-3p. Lower panel, the luciferase reporter plasmids containing wild type (WT) or mutant (MUT) MK5-AS1 were cotransfected into HCT116 cells with let-7f-1-3p. **f, g.** CCK8 assays and colony formations were used to determine the proliferation of HCT116 and SW620 after transfection. The data represented the mean ± SD from three independent experiments. *P < 0.05, **P < 0.01 and ***P < 0.001

**Fig. 7:**
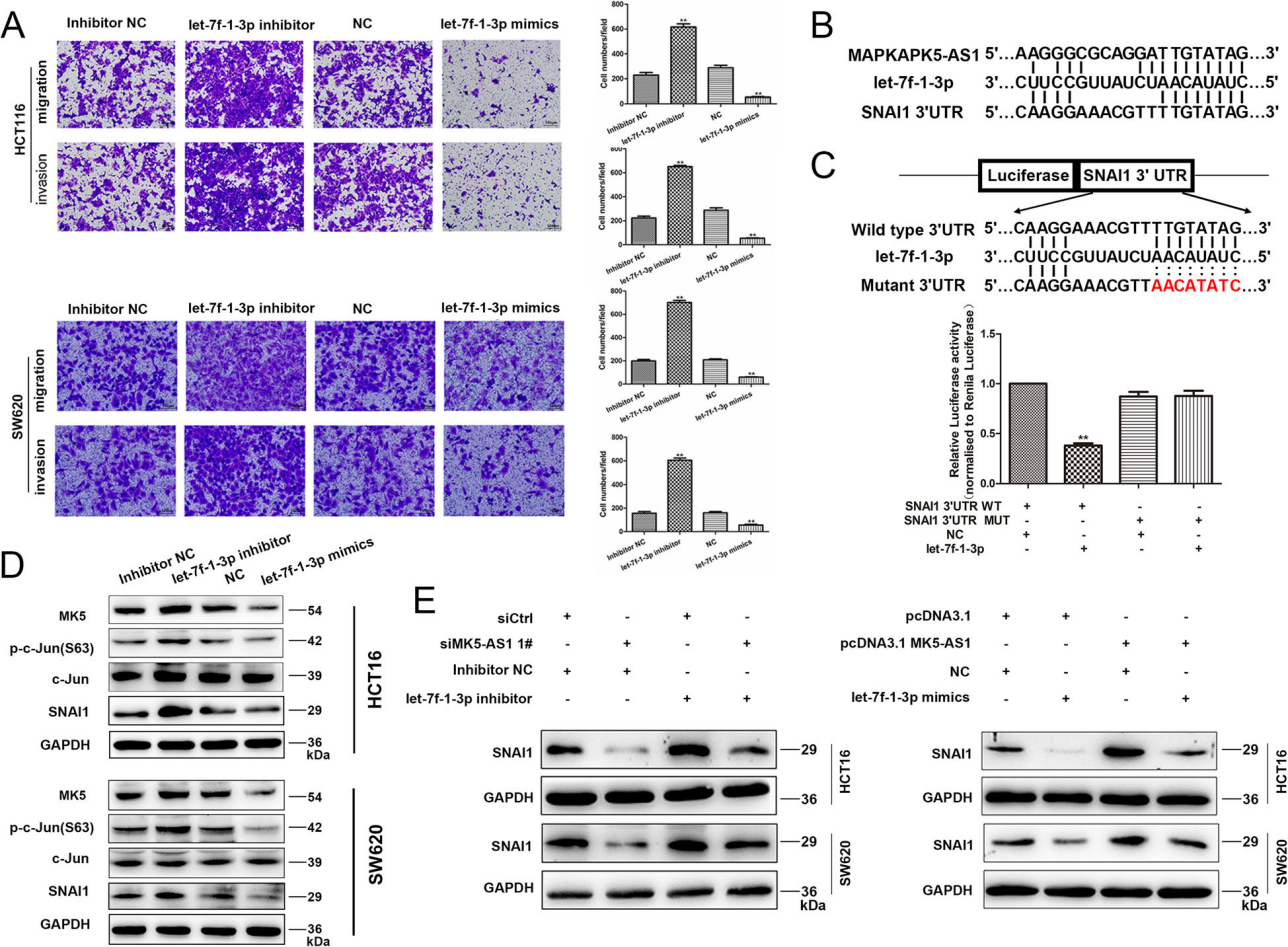
MK5-AS1/let-7f-1-3p/SNAI1 ceRNA network. **a.** Transwell assays were used to perform the invasion and migration abilities in HCT116 and SW620 cells after transfection. Scale bar, 100 μm for A. **b.** The potential binding sites among MK5-AS1, let-7f-1-3p and SNAI1. **c.** Upper panel, the potential binding sites between let-7f-1-3p and SNAI1. Lower panel, the luciferase reporter plasmids containing wild type (WT) or mutant (MUT) SNAI1 3′ UTR were cotransfected into HCT116 cells with let-7f-1-3p. **d.** MK5, c-Jun, p-c-Jun(S63) and SNAI1 expressions were detected in HCT116 and SW620 cells by immunoblotting after transfection with let-7f-1-3p inhibitor or let-7f-1-3p mimics. **e.** Left panel, the effects of si-MK5-AS1, let-7f-1-3p inhibitor and si-MK5-AS1 + let-7f-1-3p inhibitor on protein levels of SNAI1 in HCT116 and SW620 cells. Right panel, the effects of pcDNA3.1 MK5-AS1, let-7f-1-3p mimics, and pcDNA3.1 MK5-AS1 + let-7f-1-3p mimics on protein levels of SNAI1 in HCT116 and SW620 cells. Data were shown as mean ± SD for three independent experiments. *P < 0.05, **P < 0.01 and ***P < 0.001

As mentioned above, MK5-AS1 was mainly located in the cytoplasm and played a major role in posttranscriptional regulation. To test the hypothesis that MK5-AS1 functions as a ceRNA in regulating mRNA expression, we used the publicly available bioinformatics databases DIANA Tools and miRcode and observed that the MK5-AS1 sequence contained potential miR-1284, let-7f-1-3p, let-7b-3p, miR-506-5p, and miR-490-5p binding sites (Fig. 6a). Previous reports have proven that the let-7 miRNA family functions as a tumor suppressor gene in human CRC and that let-7f-1-3p is downregulated in tumor tissues [33, 34]. Remarkably, MK5-AS1 overexpression inhibited miR-1284 and let-7f-1-3p expression, while MK5-AS1 knockdown increased the expression level of let-7f-1-3p but not that of miR-1284 (Fig. 6b). Furthermore, we used qPCR to detect the expression level of let-7f-1-3p in 42 pairs of matched colorectal tissues and corresponding normal tissues (Fig. 6c). The data revealed that let-7f-1-3p downregulated in CRC tissues compared with normal tissues, and an inverse correlation between MK5-AS1 and let-7f-1-3p expression was observed (Fig. 6d). We generated a reporter construct in which the predicted binding site of let-7f-1-3p in the MK5-AS1 sequence was mutated by site-specific mutagenesis, which, as expected, eliminated the let-7f-1-3p-mediated inhibition of luciferase activity (Fig. 6e). Consequently, we selected let-7f-1-3p as a candidate for further research and transfected HCT116 and SW620 cells with let-7f-1-3p inhibitor or let-7f-1-3p mimics. We then performed CCK8 and colony formation assays and found that silencing of let-7f-1-3p enhanced cell proliferation and colony formation, and overexpression of let-7f-1-3p had the opposite effects (Fig. 6f and g). Transwell assays demonstrated that cell migration and invasion were induced by silencing let-7f-1-3p expression and were clearly decreased by overexpression of let-7f-1-3p (Fig. 7a).

According to the sequence analysis of SNAI1, the 3′ UTR of SNAI1 (748–771 nucleotides) contains a let-7f-1-3p binding site. Our previous results confirmed that MK5-AS1 upregulated the expression of SNAI1, so we speculated that MK5-AS1 acted as a ceRNA to regulate SNAI1 by sponging let-7f-1-3p (Fig. 7b). Subsequently, we performed a luciferase reporter assay to validate the binding of let-7f-1-3p with SNAI1. The results demonstrated that overexpression of let-7f-1-3p markedly reduced the luciferase activity of the reporter containing the predicted let-7f-1-3p binding site (wt-SNAI1), but this inhibition was abolished by mutation of the predicted let-7f-1-3p binding site in the SNAI1 3′ UTR (mut-SNAI1) in HCT116 cells (Fig. 7c). To test whether SNAI1 was regulated by let-7f-1-3p in CRC cells, we measured the levels of MK5, p-c-Jun, c-Jun and SNAI1 when let-7f-1-3p was suppressed or overexpressed in HCT116 and SW620 cells. We found that inhibition of let-7f-1-3p promoted the phosphorylation of c-Jun S63 site and the expression of MK5 and SNAI1, overexpression of let-7f-1-3p decreased the phosphorylation of c-Jun S63 site and the expression of MK5 and SNAI1 (Fig. 7d and Fig. S4C).

Next, we wanted to verify whether MK5-AS1 could regulate SNAI1 by targeting let-7f-1-3p in CRC cells via cotransfection of cells with si-MK5-AS1 #1 and let-7f-1-3p inhibitor. Interestingly, the inhibition of SNAI1 protein expression induced by si-MK5-AS1 #1 was effectively reversed by let-7f-1-3p inhibitor. In addition, the level of SNAI1 protein increased after MK5-AS1 overexpression, which was reversed when pcDNA3.1 MK5-AS1 and let-7f-1-3p mimics were cotransfected (Fig. 7e). In summary, the above results suggested that MK5-AS1 regulated the expression of SNAI1 by sponging let-7f-1-3p.

## Discussion

Recently, emerging evidence has suggested that lncRNAs play a vital role in cellular development and human diseases, especially in cancer [12]. Dysregulated expression of lncRNAs might lead to progressive, uncontrolled growth and metastasis of tumors [14, 35]. Using a combination of genomic, biochemical, and cell biological analysis, we proved that MK5-AS1 is an oncogenic lncRNA in CRC. Database and clinical specimen analysis demonstrated that MK5-AS1 was upregulated in CRC and induced cell proliferation and metastasis. Moreover, a change in MK5-AS1 levels was correlated with advanced TNM stage, including lymph node metastasis, peripheral metastasis, pathological staging, tumor development and tumorigenesis, which is consistent with previous reports [27]. However, Wang et al. mainly focused on MK5-AS1 enhancing CRC development by targeting p21 [27], and the mechanism of MK5-AS1 promoting tumor metastasis and invasion was poorly defined. MK5-AS1 is an antisense transcript of MK5, and both genes share overlapping sequences in the promoter and first exon region, so MK5-AS1 could *cis*-regulate the neighboring gene MK5. The MK5 gene has been proven to have dual characteristics in cancer progression [36], hence, the effects of high expression of MK5 on OS in the TCGA database were in contradiction with ours. MK5-AS1 was found to be mainly located in the cytoplasm, which did not correspond with a previous study [27] of MK5-AS1 showing that it was mainly located in the nucleus. However, the results do not disapprove MK5-AS1 as having a nonrestrictive subcellular localization. This discrepancy may be due to the heterogeneity of tumor cells or differences in the activation status. Cytoplasmic lncRNAs might participate in modulating the translation process and regulating RNA and protein stability and modification; however, the underlying molecular mechanisms of MK5-AS1 remain unknown.

**Fig. 8:**
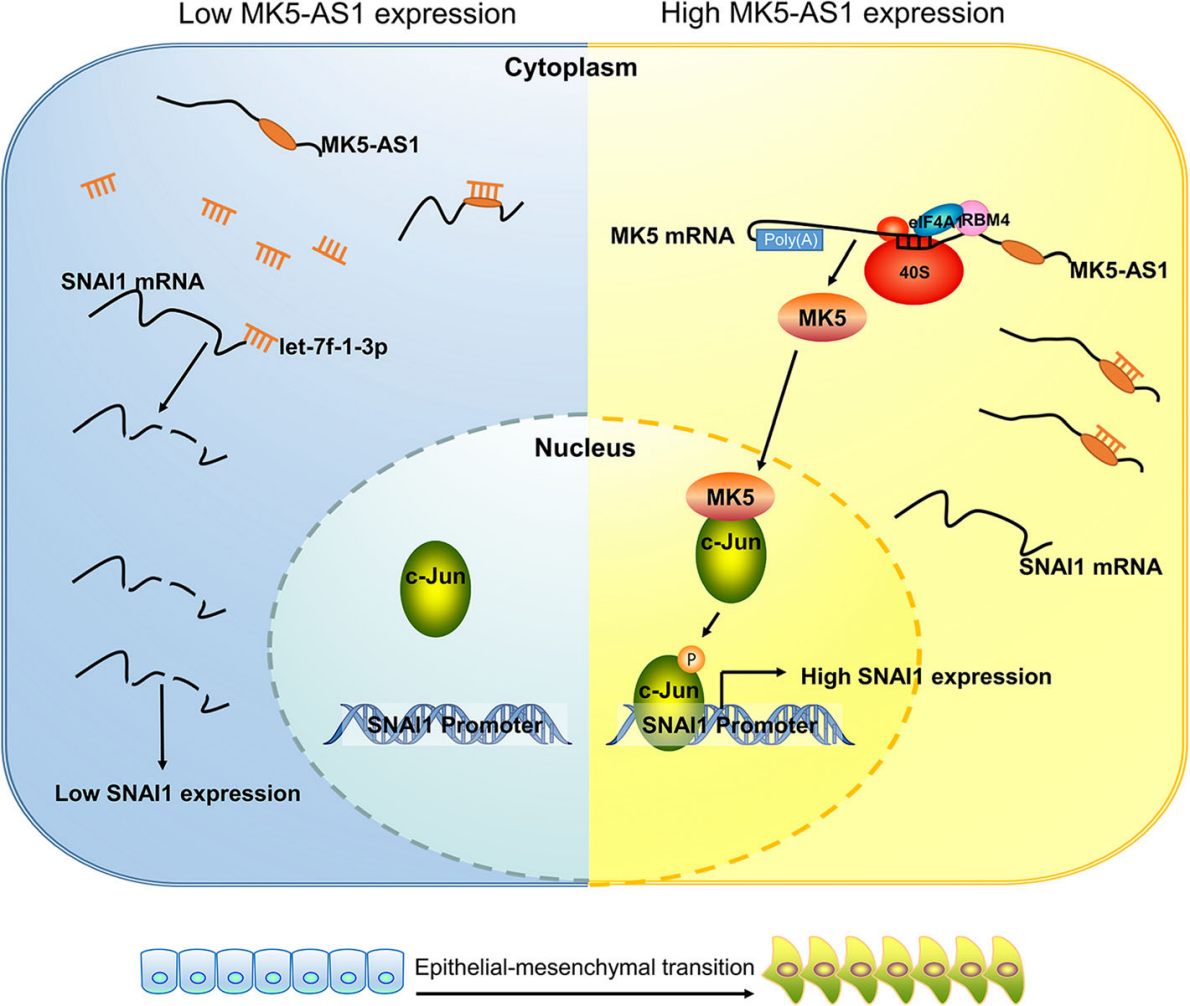
A hypothetical model depicts the roles of MK5-AS1 in the promotion of EMT in CRC

In addition, we observed that MK5-AS1 could positively regulate SNAI1 and MK5 mRNA and protein expression. Over the last decade, research studies have focused on inhibiting or even reversing SNAI1-induced EMT, including a series of cell morphological conversions, changes in extracellular matrix properties, and redistribution of surface markers on membranes [37, 38]. Nevertheless, most trials with SNAI1 inhibitors have not been optimistic, which could be attributed, at least in part, to suboptimal target selection. Subsequently, inhibition of SNAI1 before being translated into mature protein was more significant in restraining metastasis. Mechanistically, MK5-AS1 regulated SNAI1-mediated EMT through at least two pathways: MK5-AS1, acting as a ceRNA, promoted the levels of SNAI1 by inhibiting let-7f-1-3p expression and MK5-AS1 recruited eIF4A1, the RBM4 complex and then *cis*-regulated the neighboring gene MK5. MK5 phosphorylated the substrate protein c-Jun, which activated the transcription factor SNAI1 (Fig. 8).

Due to cytoplasmic localization of MK5-AS1, we hypothesized that MK5-AS1 promoted MK5 translation or enhanced its mRNA stability. We first demonstrated that MK5-AS1 could directly bind to RBM4, which was confirmed to interact with eIF4A1, a subunit of the protein complex eIF4F [39]. RBM4 and the eIF4A1 complex mediate the recruitment of ribosomes to mRNAs and participate in eukaryotic translation initiation [32]. Collectively, these data demonstrated that MK5-AS1 promoted MK5 expression by binding to MK5 mRNA through overlapping sequences, recruiting RBM4 and the eIF4A1 protein complex, and further initiating IRES-dependent translation initiation. Moreover, our report was in agreement with previous investigation results showing increased stability of the MK5 mRNA 5′ end and reduced degradation [40]. In our studies, MK5 promoted tumor development and metastasis, which was inconsistent with previous reports [41, 42], indicating the heterogeneity and complexity of tumor [36]. MK5 is a p38 MAP kinase-activated protein kinase that has been demonstrated by in vitro kinase assays to phosphorylate the substrate c-Jun (1–93), [43]. In light of the above information, we confirmed by co-IP and IB that MK5 could specifically phosphorylate c-Jun at the S63 site, which played a vital role in enhancing the activity of the transcription activator [44]. MK5 has been verified to contain NLS and NES sequences and to be shuttled between the cytoplasm and the nucleus [45], it is possible that MK5 could translocate into the nucleus to phosphorylate the nuclear transcription factor c-Jun. It has also been reported that there are multiple c-Jun binding sites on the SNAI1 promoter. Previous studies mentioned that the p38 MAPK pathway could regulate SNAI1 by c-Jun under cell stress conditions, and the underlying mechanism is unclear [46, 47]. In our current study, we identified a novel c-Jun binding site on the SNAI1 promoter, suggesting that c-Jun is a strong transcriptional activator of the SNAI1 promoter. Consequently, our study clearly determined that p38 MAP kinase-activated MK5 could trigger the activity of c-Jun through phosphorylation of c-Jun, which then bound to the SNAI1 promoter to promote SNAI1-mediated EMT.

It has been reported that altering extracellular responses and intracellular signal transduction, such as enhancing the activity of p38MAPK [48], JNK [49] and eIF4 [39] signaling pathways, leads to carcinogenesis and aggravates metastasis. The p38MAPK pathway responds and adapts to numerous extracellular stimuli through downstream targets, which include protein kinases, phosphatases, transcription factors, and cell-cycle regulators. It has come to light that c-Jun participates in the JNK and stress-activated MAPK signaling pathways [50]. MK5, which is directly phosphorylated by MAPK4/6 (Erk3/4), could be phosphorylated and catalyzed by p38 MAP kinase instead of JNK [51]. Additionally, MK5 has been confirmed to phosphorylate the substrate eIF4E [43], and MK5-AS1 was analyzed as a possible direct binding partner of eIF4B (predicted by RBPDB). Consequently, the functional interaction between MK5-AS1 and MK5 appears to be involved in multiple positive feedback loops in regulating MK5 translation to facilitate the oncogenic phenotype, but this has not been verified. Furthermore, we hypothesized that MK5-AS1 in the cytoplasm could participate in RNA networks through MREs. MK5-AS1 and SNAI1 mRNA were found to be potential targets of let-7f-1-3p/miR-1284. We verified the expression of let-7f-1-3p/miR-1284 in CRC cell lines and confirmed that MK5-AS1-let-7f-1-3p-SNAI1 mRNA acted as a ceRNA. The mature sequence and seed sequence of let-7f-1-3p and miR-1284 are highly analogous, which revealed the importance of this binding site for RNA “Talk” and the similarity of their biological functions. According to our analysis, MK5-AS1 might regulate MK5 mRNA by acting as a ceRNA, as both of them possessed analogous MREs and high abundance miRNAs as the underlying targets. In addition, other highly complementary mRNAs with MK5-AS1 also exhibited potential ceRNA properties. The RNA network is a multilayered, multidirectional, multi-interactive and multidimensional network architecture that orchestrates cellular responses. Whether MK5-AS1 communicated with other microRNAs/lncRNAs/mRNAs through MREs to form RNA regulatory networks needs to be further confirmed.

## Conclusion

In conclusion, we found that MK5-AS1 functioned as a carcinogenic lncRNA during CRC progression and revealed a novel regulatory pathway in which MK5-AS1 upregulated SNAI1 expression by sponging let-7f-1-3p and the *cis*-regulating adjacent gene MK5. We identified MK5-AS1 as a cancer-associated lncRNA, which could be a novel potential therapeutic target for CRC.

## Supplementary information

**Additional file 1: Table S1.** List of primers and siRNA/shRNA sequences.

**Additional file 2: Figure S1.** MK5-AS1 promoted CRC cells proliferation. A. MK5-AS1 was detected by qPCR after MK5-AS1 knockdown in HCT116 cells. B. Overexpression of MK5-AS1 was detected by qPCR in HCT116 cells. C. CCK8 assays were utilized to determine the viability of HCT116 and SW620 cells after transfection. D. Colony formations were used to perform the proliferative capacity of CRC cells after intervening MK5-AS1, respectively. E. The RNAs levels of MK5-AS1, MK5 and SNAI1 were detected by qPCR after intervening MK5-AS1, respectively. The data represented the mean ± SD from three independent experiments. *P < 0.05, **P < 0.01 and ***P < 0.001.

**Additional file 3: Figure S2.** MK5 expression in paired human CRC tissues (T) and adjacent non-tumor tissues (N) was detected by immunoblotting. The data represented the mean ± SD from three independent experiments.

**Additional file 4: Figure S3.** MK5 promoted CRC cells proliferation. A. Kaplan-Meier overall survival was analyzed according to MK5 expression in TCGA cohort and our cohort. B. The effectiveness of MK5 intervention were detected in HCT116 cells. C. CCK8 assays were performed to determine the viability of HCT116 and SW620 cells after transfection. D. Colony formations were used to determine the proliferative capacity of HCT116 and SW620 cells after intervening MK5, respectively. The data represented the mean ± SD from three independent experiments. *P < 0.05, **P < 0.01 and ***P < 0.001.

**Additional file 5: Figure S4.** A. Identification of the c-Jun binding sequences in SNAI1 promoters by CHIP-PCR. B. The existence of SNAI1 promoter sequences with endogenous c-Jun was measured by CHIP-PCR. C. qPCR was utilized to detect the mRNA level of SNAI1 in HCT116 and SW620 cells after intervening let-7f-1-3p, respectively. The data represented the mean ± SD from three independent experiments. *P < 0.05, **P < 0.01 and ***P < 0.001.

**Additional file 6: Figure S5.** The relative expression of proteins in each group was statistically analyzed.

**Additional file 7: Figure S6.** The relative expression of proteins in each group was statistically analyzed.

**Additional file 8: Figure S7.** A, B. qPCR was utilized to analyze the MK5-AS1 expression in 172 pairs of CRC tissues and corresponding adjacent non-tumoral tissues.

**Additional file 9.**

## Data Availability

All data generated or analyzed during the current study are available from the corresponding author on reasonable request.

## Abbreviations

lncRNAs: Long noncoding RNAs
CRC: Colorectal cancer
qPCR: Quantitative real-time PCR
CCK8: Cell Counting Kit-8
RIP: RNA immunoprecipitation
ChIP: Chromatin immunoprecipitation
HOTAIR: HOX transcript antisense RNA
UICLM: Upregulated in colorectal cancer liver metastasis
EMT: Epithelial-mesenchymal transformation
ceRNAs: Competitive endogenous RNAs
MREs: miRNA response elements
DMEM: Dulbecco’s modified Eagle medium
si-NC: Negative control siRNA
OD: Optical density
co-IP: Co-immunoprecipitation
RNA-FISH: RNA-Fluorescence in situ hybridization
OS: Overall survival
MK5: MAPK activated protein kinase 5
