# Correction: Long noncoding RNA MAPKAPK5-AS1 promotes colorectal cancer progression by cis-regulating the nearby gene MK5 and acting as a let-7f-1-3p sponge

**DOI:** 10.1186/s13046-022-02537-5

**Published:** 2022-12-13

**Authors:** Ting Yang, Wei-Cong Chen, Pei-Cong Shi, Man-Ru Liu, Tao Jiang, Hu Song, Jia-Qi Wang, Rui-Zhi Fan, Dong-Sheng Pei, Jun Song

**Affiliations:** 1 Department of General Surgery, The Affiliated Hospital of Xuzhou Medical University, Xuzhou, 221002 Jiangsu Province China; 2 Department of Pathology, Xuzhou Medical University, Xuzhou, 221002 Jiangsu Province China; 3 Institute of Digestive Diseases of Xuzhou Medical University, Xuzhou, 221002 Jiangsu Province China

**Correction:** ***J Exp Clin Cancer Res* 39, 139 (2020)**

**https://doi.org/10.1186/s13046-020-01633-8**

Following the publication of the original article [1], authors found that several images of the “Transwell assay” in Figs. 2A, 3E and 7A contained an inter-duplication. They carefully checked all the original data in this study and found that some images were misused in different group and have replaced them with the corrected images.

The authors regret for the inadvertent errors that have been made in preparation of the published figures, and confirm that these errors did not affect the conclusions or discussion of the article. The authors apologize for any inconvenience caused by this mistake.

The corrected figures are also provided below. The original article has been corrected.

**Fig. 2:**
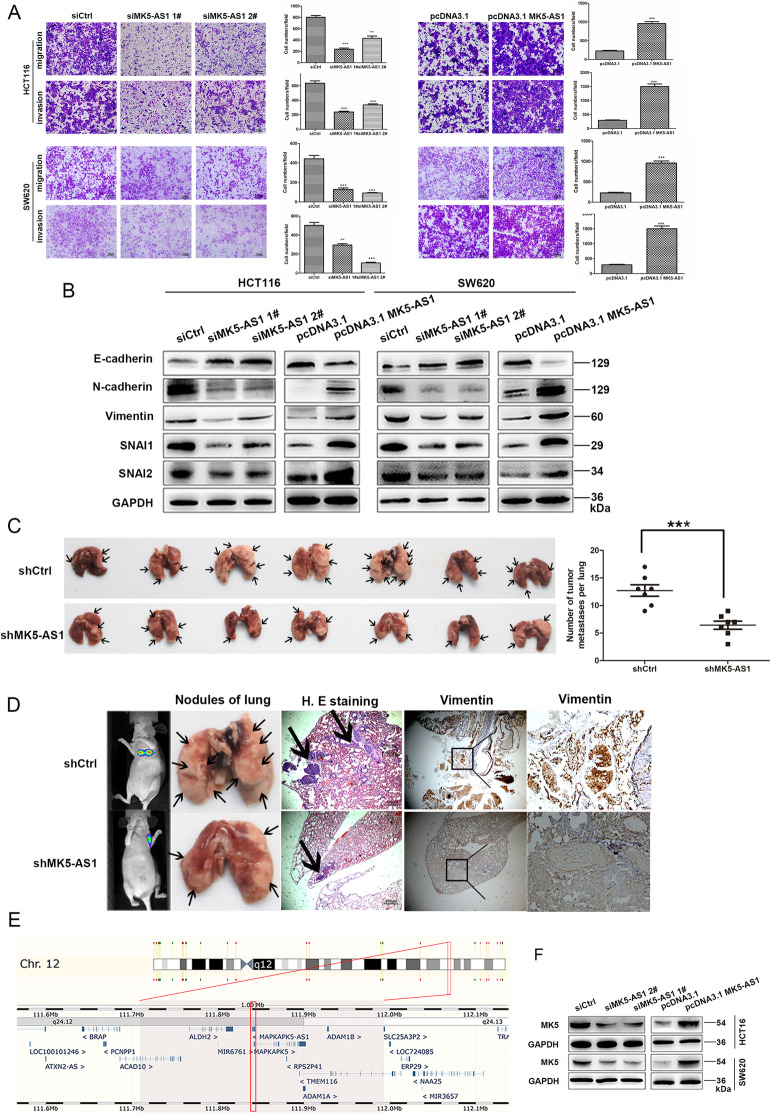
MK5-AS1 regulated CRC invasion and migration in vitro and *vivo*. **a** Transwell assays were used to determine the invasion and migration abilities of HCT116 and SW620 cells after MK5-AS1 overexpression and knockdown. Scale bar, 100 μm for A. **b** Immunoblotting of EMT-related markers after transfection in CRC cells. **c** Nude mice were injected with HCT116 cell after MK5-AS1 knockdown into tail vein. The number of metastatic nodules of lung was shown and counted. The black arrow marked metastatic nodules. **d** Tumor progression was monitored using a small animal imaging system, HE-stained lung sections (Scale bar, 100 μm) and antibody vimentin of metastatic nodules were shown (× 400 magnification). The black arrow marked metastatic nodules. **e** The Ensembl Genome browser (http://asia.ensembl.org/) showed that MK5 was the nearby gene of MK5-AS1. **f** Immunoblotting was used to investigate the level of MK5 of HCT116 and SW620 cells after intervening MK5-AS1, respectively. The data represented the mean ± SD from three independent experiments. *P < 0.05, **P < 0.01 and ***P < 0.001

**Fig. 3:**
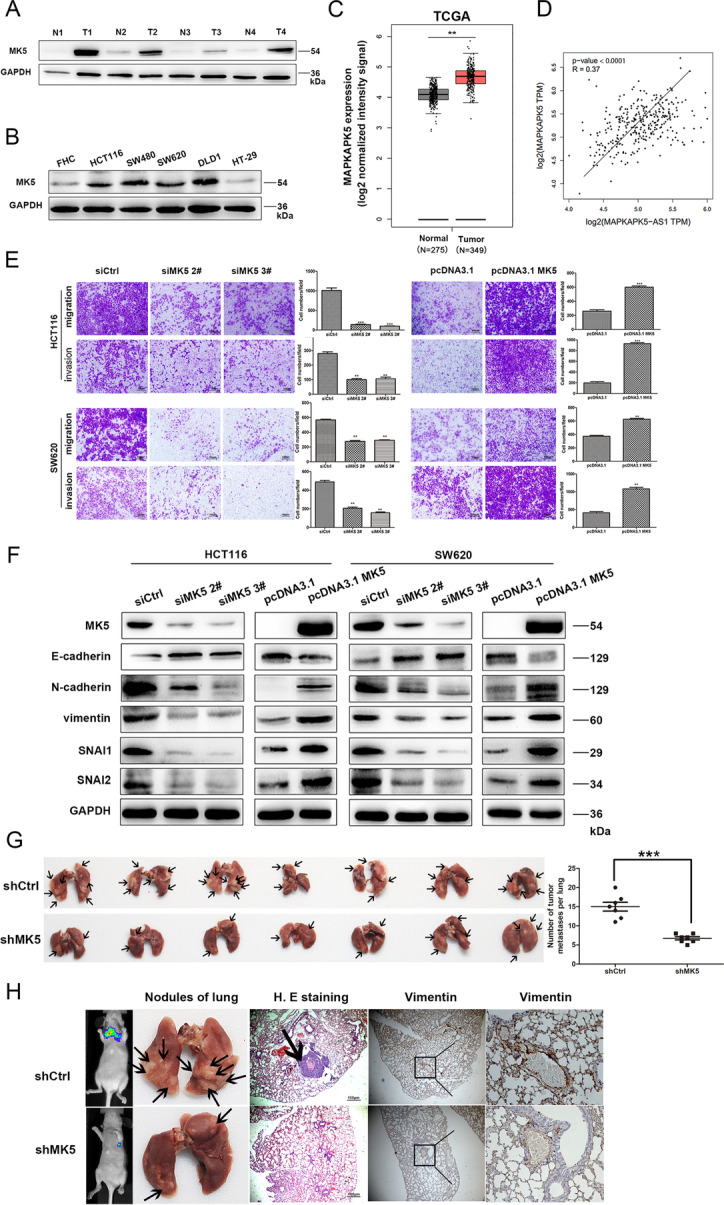
MK5 promoted CRC cells invasion and migration in vitro and *vivo*. **a** Immunoblotting of MK5 expression in 4 pairs of human CRC tissues (T) and adjacent non-tumor tissues (N). **b** Expression of MK5 in the normal colorectal epithelium cell line (FHC) and CRC cells by immunoblotting. **c** Statistical analysis of MK5 expression in TCGA database. **d** Correlation analysis of the expression of MK5-AS1 and MK5 in GEPIA. **e** Transwell assays were used to determine the changes in invasion and migration abilities of HCT116 and SW620 cells after transfection. Scale bar, 100 μm for E. **f** Immunoblotting analysis of EMT-related markers after transfection in CRC cells, respectively. **g** The number of metastatic pulmonary nodules was shown and counted. The black arrow marked metastatic nodules. **h** After HCT116 cells with knockdown MK5 were injected into the tail vein of nude mice, In vivo fluorescence imaging, the gross lesion in lung tissues and H. E staining and antibody vimentin of metastatic nodules in the lungs were observed (× 400 magnification). Scale bar, 100 μm for H.E. The black arrow marked metastatic nodules. The data represented the mean ± SD from three independent experiments. *P < 0.05, **P < 0.01 and ***P < 0.001

**Fig. 7:**
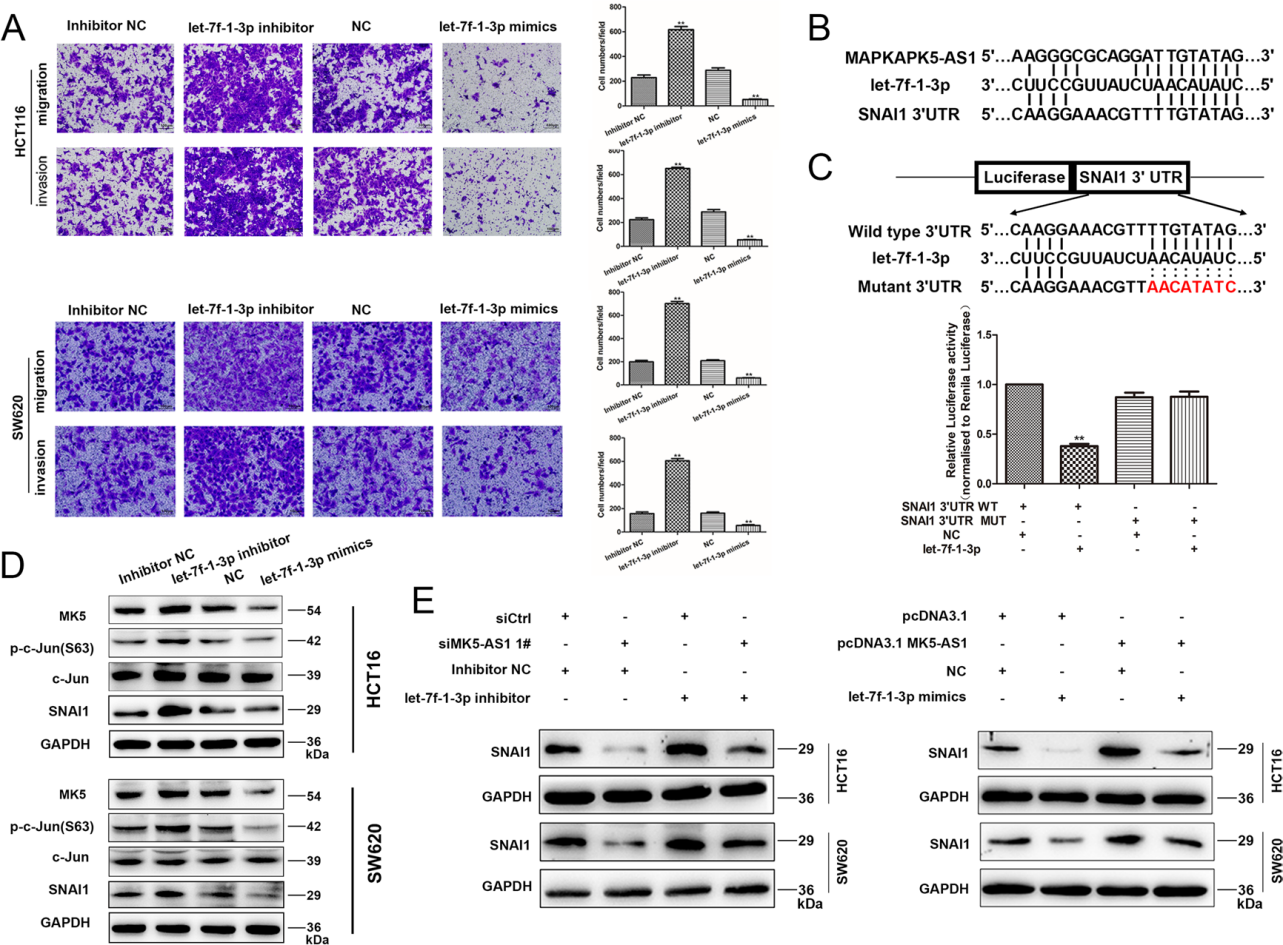
MK5-AS1/let-7f-1-3p/SNAI1 ceRNA network. **a** Transwell assays were used to perform the invasion and migration abilities in HCT116 and SW620 cells after transfection. Scale bar, 100 μm for A. **b** The potential binding sites among MK5-AS1, let-7f-1-3p and SNAI1. **c** Upper panel, the potential binding sites between let-7f-1-3p and SNAI1. Lower panel, the luciferase reporter plasmids containing wild type (WT) or mutant (MUT) SNAI1 3′ UTR were cotransfected into HCT116 cells with let-7f-1-3p. **d** MK5, c-Jun, p-c-Jun(S63) and SNAI1 expressions were detected in HCT116 and SW620 cells by immunoblotting after transfection with let-7f-1-3p inhibitor or let-7f-1-3p mimics. **e** Left panel, the effects of si-MK5-AS1, let-7f-1-3p inhibitor and si-MK5-AS1 + let-7f-1-3p inhibitor on protein levels of SNAI1 in HCT116 and SW620 cells. Right panel, the effects of pcDNA3.1 MK5-AS1, let-7f-1-3p mimics, and pcDNA3.1 MK5-AS1 + let-7f-1-3p mimics on protein levels of SNAI1 in HCT116 and SW620 cells. Data were shown as mean ± SD for three independent experiments. *P < 0.05, **P < 0.01 and ***P < 0.001
