# Supplementary figures and images for: Long noncoding RNA MAPKAPK5-AS1 promotes colorectal cancer progression by cis-regulating the nearby gene MK5 and acting as a let-7f-1-3p sponge
Source: J Exp Clin Cancer Res. 2020 Jul 20;39:139. doi: 10.1186/s13046-020-01633-8 (PMC7370515; doi:10.1186/s13046-020-01633-8)

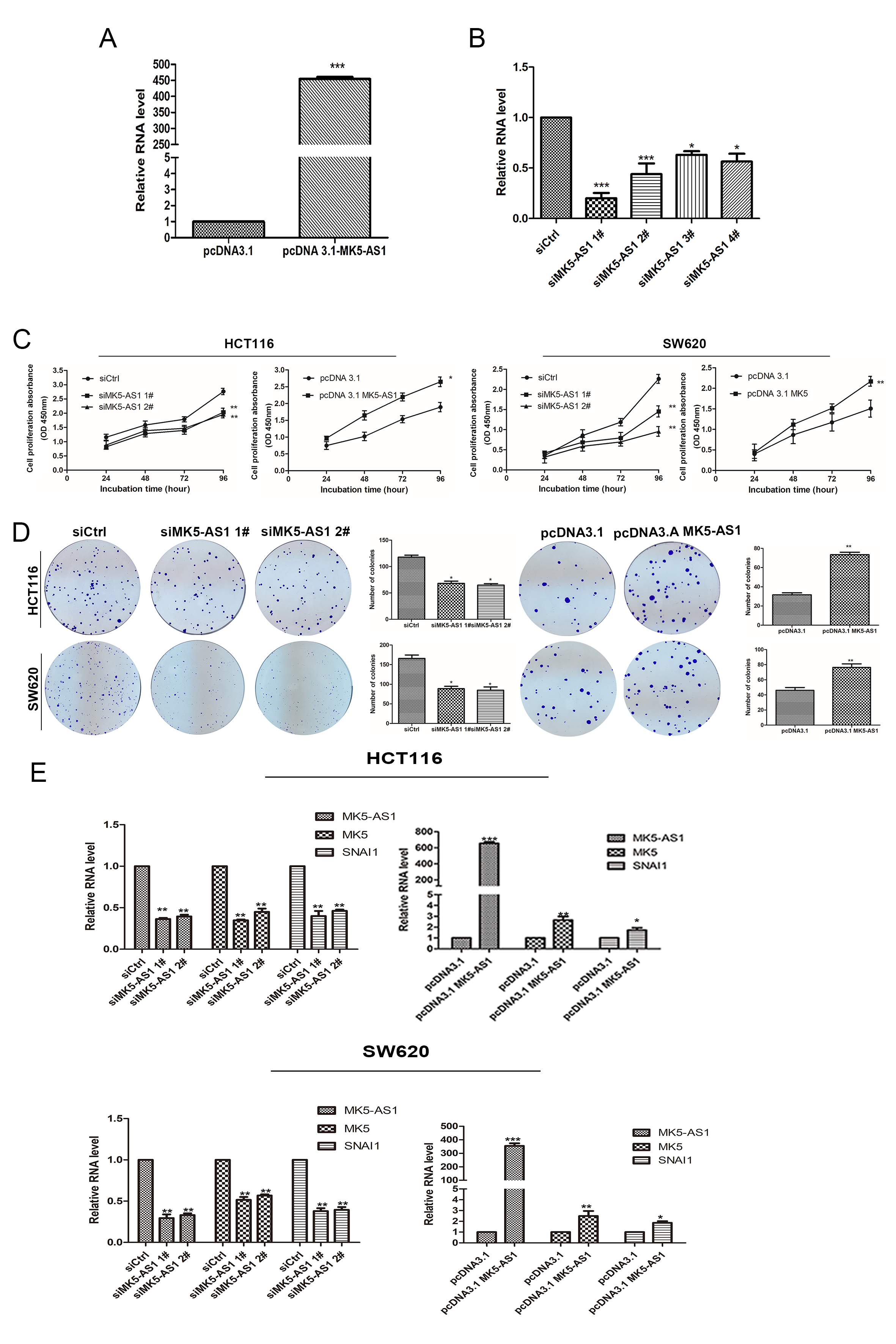

Supplement: Supplementary file 2 — Additional file 2: Figure S1. MK5-AS1 promoted CRC cells proliferation. A. MK5-AS1 was detected by qPCR after MK5-AS1 knockdown in HCT116 cells. B. Overexpression of MK5-AS1 was detected by qPCR in HCT116 cells. C. CCK8 assays were utilized to determine the viability of HCT116 and SW620 cells after transfection. D. Colony formations were used to perform the proliferative capacity of CRC cells after intervening MK5-AS1, respectively. E. The RNAs levels of MK5-AS1, MK5 and SNAI1 were detected by qPCR after intervening MK5-AS1, respectively. The data represented the mean ± SD from three independent experiments. *P < 0.05, **P < 0.01 and ***P < 0.001. [file 13046_2020_1633_MOESM2_ESM.tif]

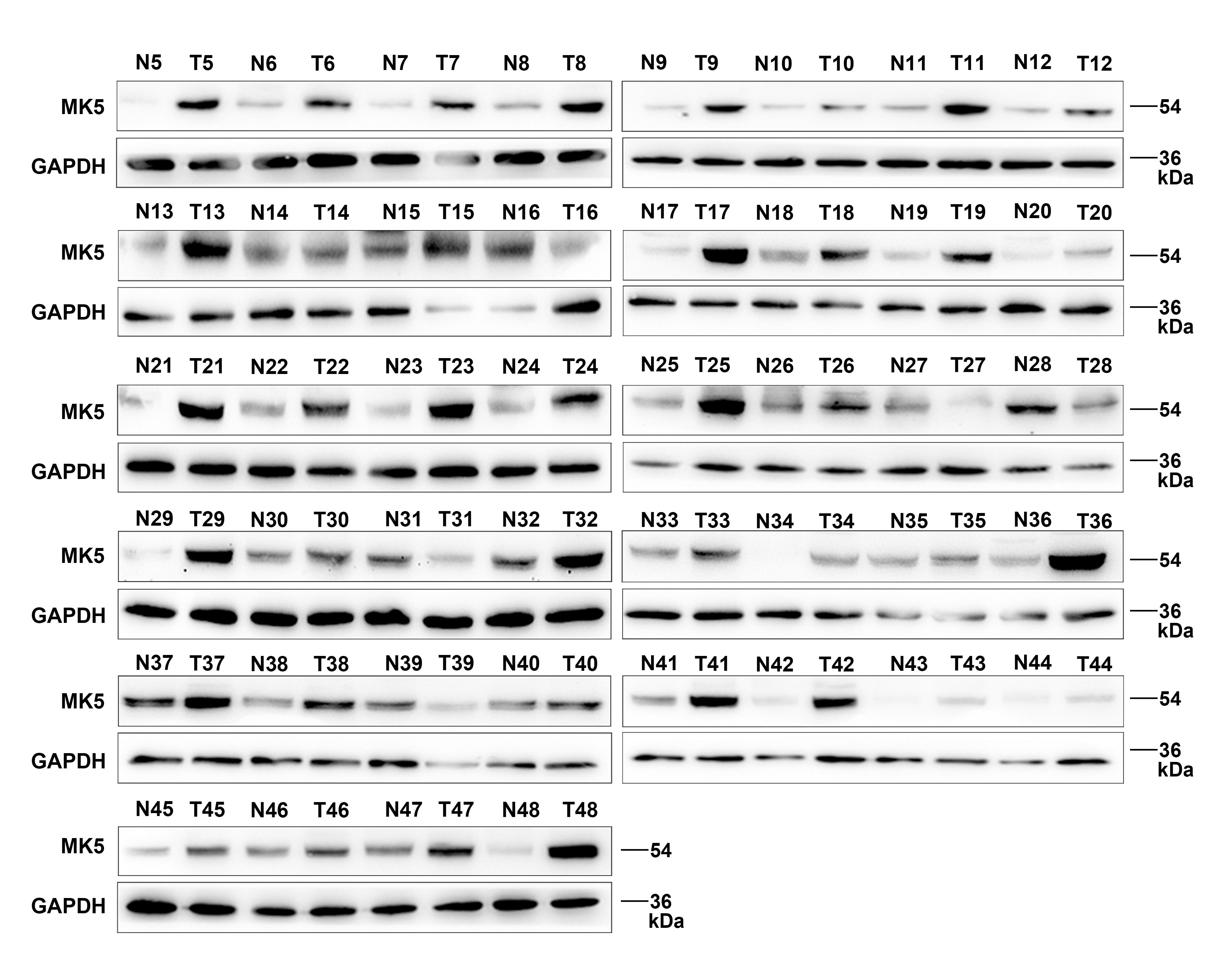

Supplement: Supplementary file 3 — Additional file 3: Figure S2. MK5 expression in paired human CRC tissues (T) and adjacent non-tumor tissues (N) was detected by immunoblotting. The data represented the mean ± SD from three independent experiments. [file 13046_2020_1633_MOESM3_ESM.tif]

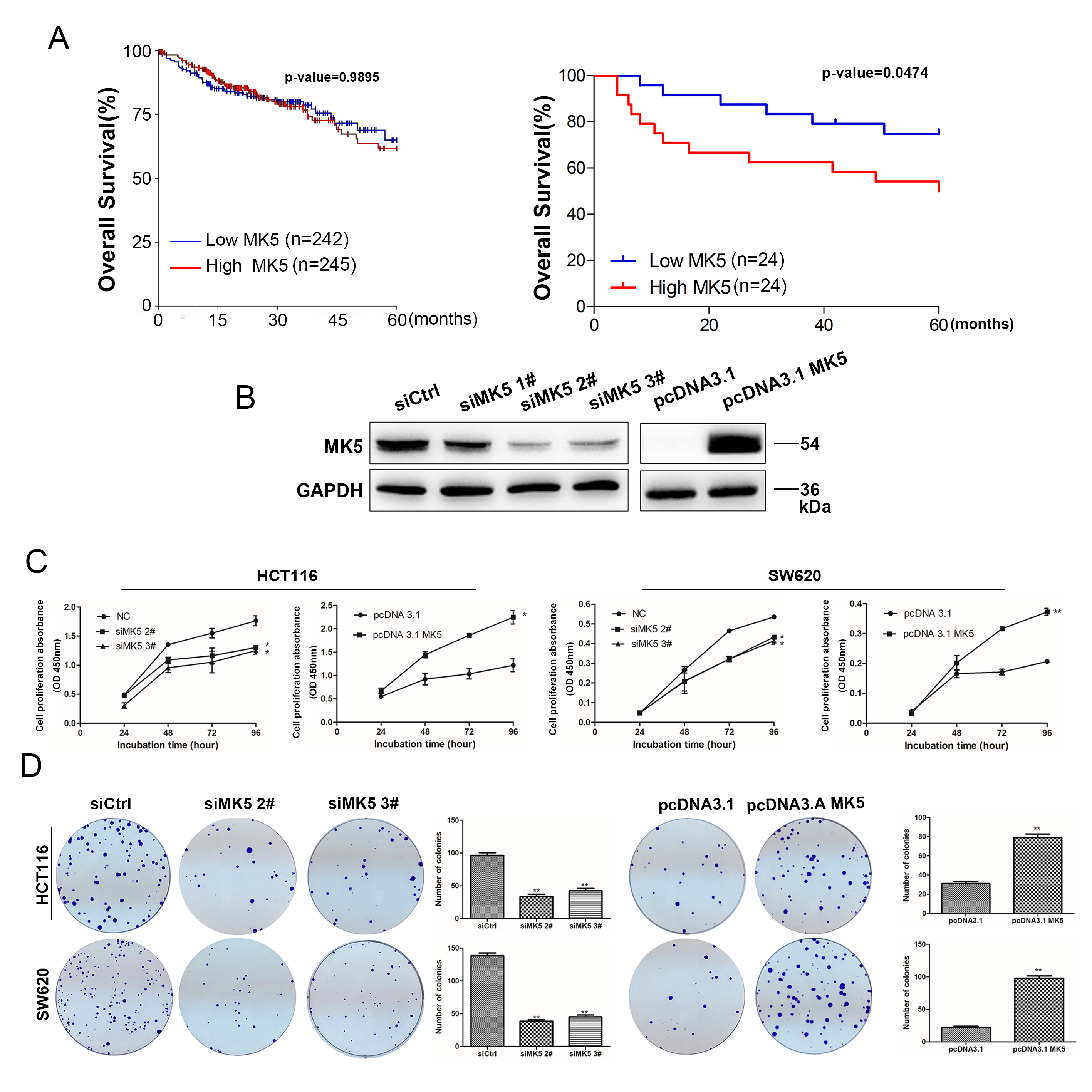

Supplement: Supplementary file 4 — Additional file 4: Figure S3. MK5 promoted CRC cells proliferation. A. Kaplan-Meier overall survival was analyzed according to MK5 expression in TCGA cohort and our cohort. B. The effectiveness of MK5 intervention were detected in HCT116 cells. C. CCK8 assays were performed to determine the viability of HCT116 and SW620 cells after transfection. D. Colony formations were used to determine the proliferative capacity of HCT116 and SW620 cells after intervening MK5, respectively. The data represented the mean ± SD from three independent experiments. *P < 0.05, **P < 0.01 and ***P < 0.001. [file 13046_2020_1633_MOESM4_ESM.tif]

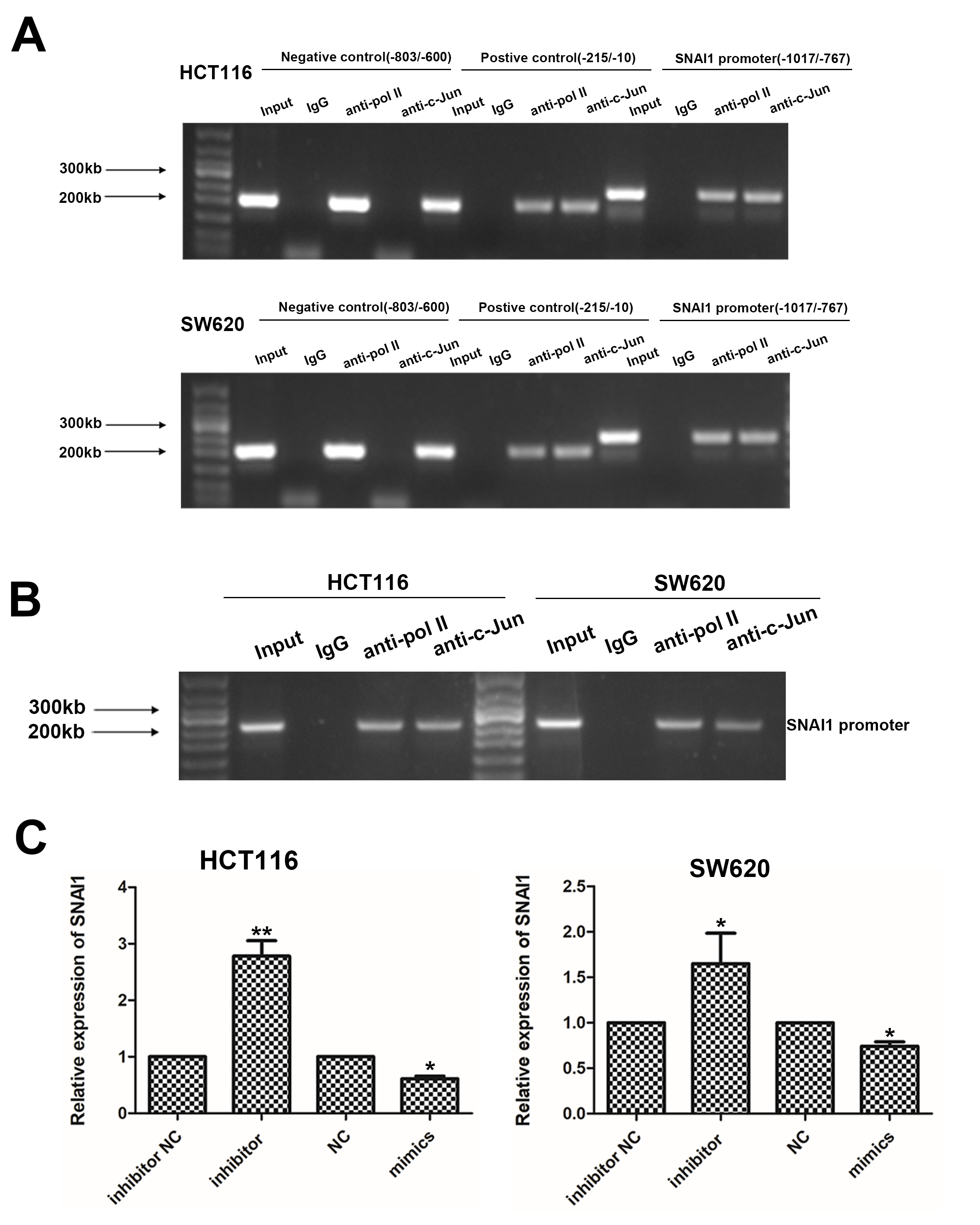

Supplement: Supplementary file 5 — Additional file 5: Figure S4. A. Identification of the c-Jun binding sequences in SNAI1 promoters by CHIP-PCR. B. The existence of SNAI1 promoter sequences with endogenous c-Jun was measured by CHIP-PCR. C. qPCR was utilized to detect the mRNA level of SNAI1 in HCT116 and SW620 cells after intervening let-7f-1-3p, respectively. The data represented the mean ± SD from three independent experiments. *P < 0.05, **P < 0.01 and ***P < 0.001. [file 13046_2020_1633_MOESM5_ESM.tif]

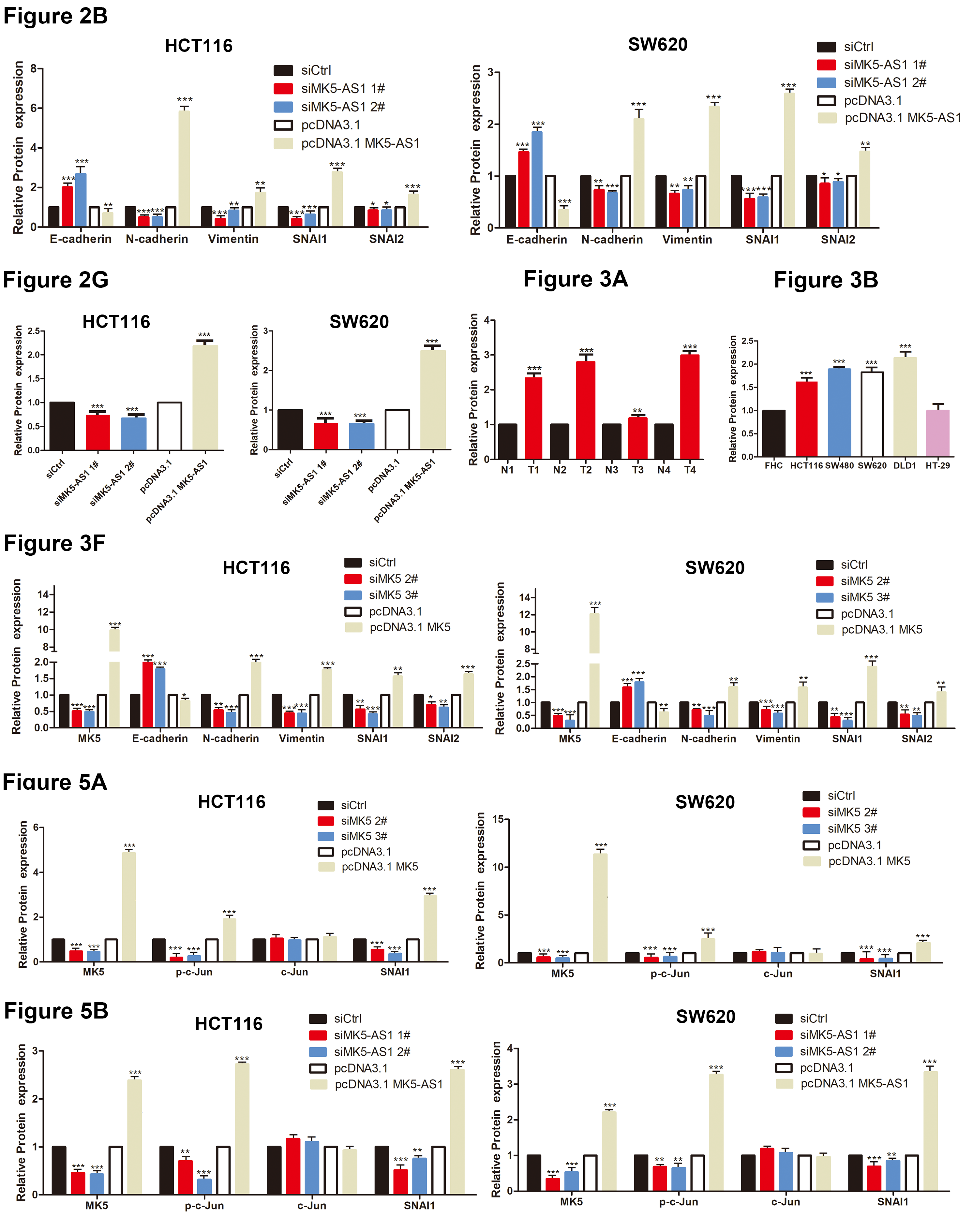

Supplement: Supplementary file 6 — Additional file 6: Figure S5. The relative expression of proteins in each group was statistically analyzed. [file 13046_2020_1633_MOESM6_ESM.tif]

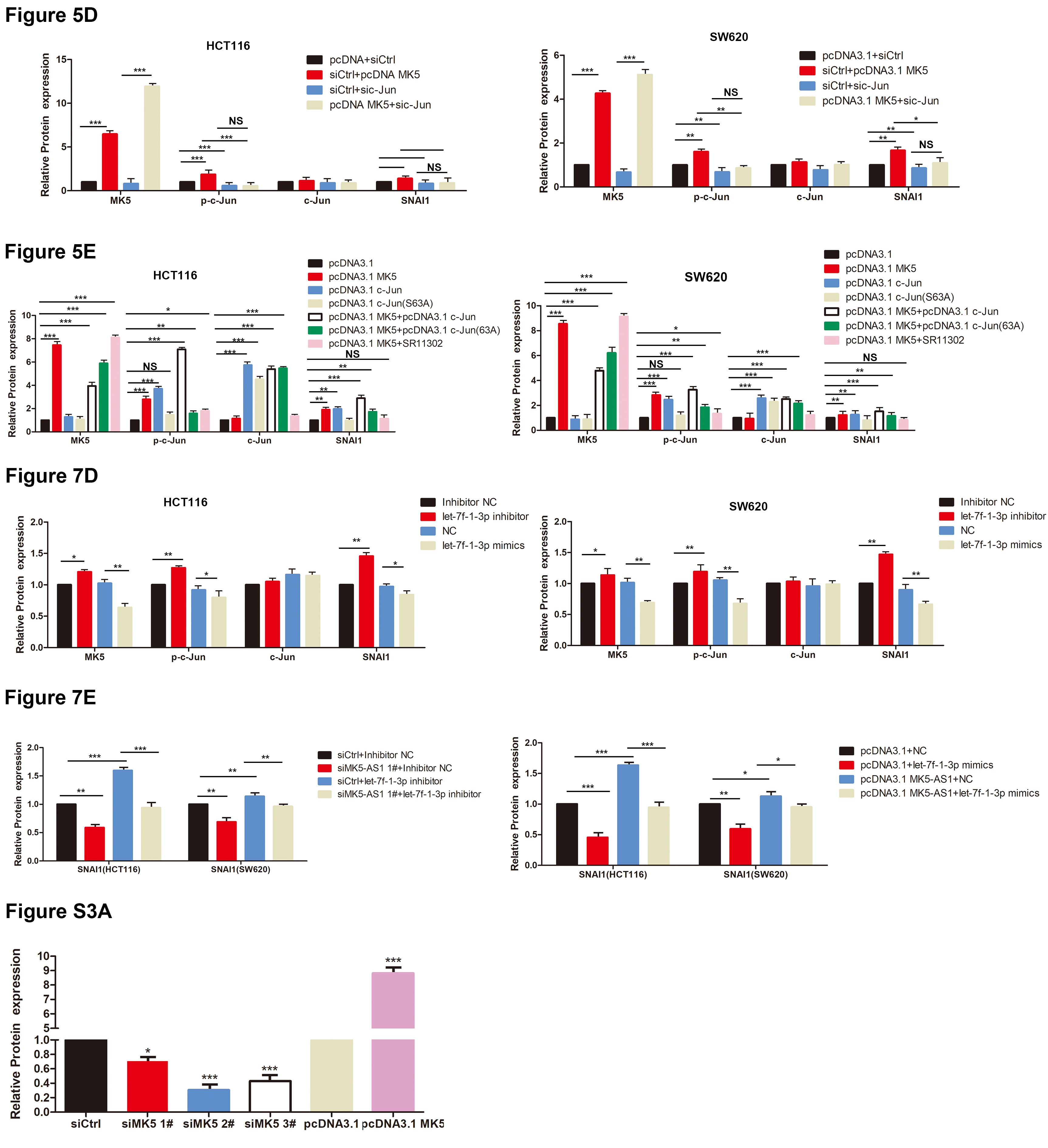

Supplement: Supplementary file 7 — Additional file 7: Figure S6. The relative expression of proteins in each group was statistically analyzed. [file 13046_2020_1633_MOESM7_ESM.tif]

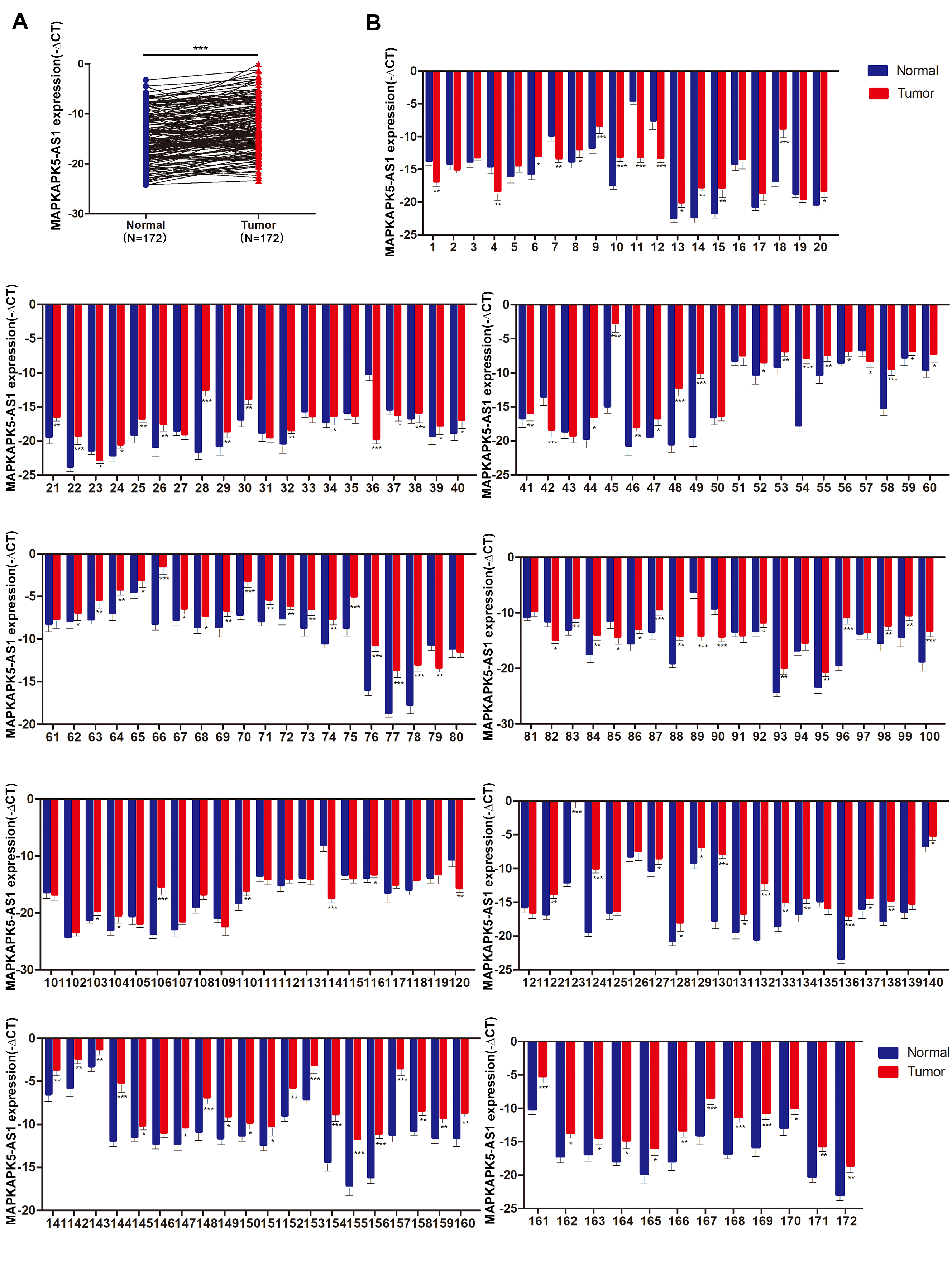

Supplement: Supplementary file 8 — Additional file 8: Figure S7. A, B. qPCR was utilized to analyze the MK5-AS1 expression in 172 pairs of CRC tissues and corresponding adjacent non-tumoral tissues. [file 13046_2020_1633_MOESM8_ESM.tif]

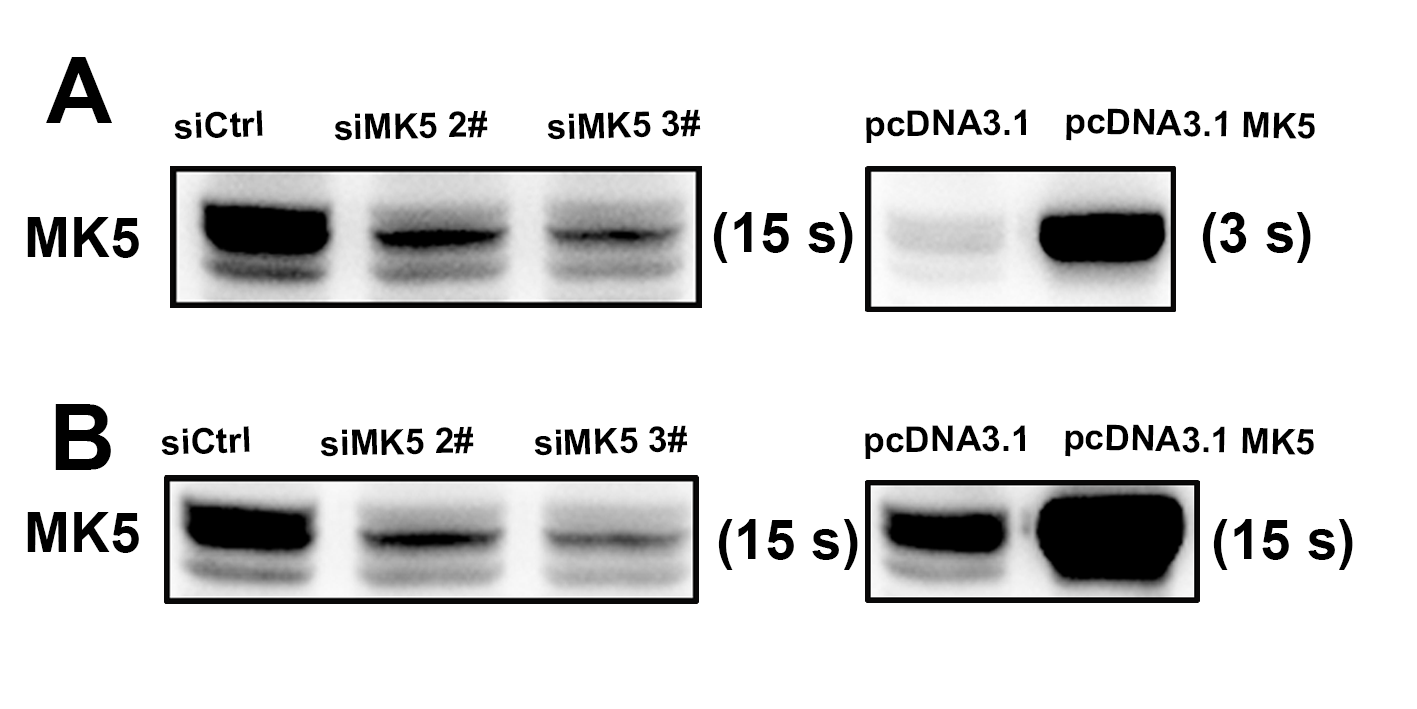

Supplement: Supplementary file 9 — Additional file 9. [file 13046_2020_1633_MOESM9_ESM.tif]
